# Ectopic expression of wax ester synthase under a wood-specific promoter enhances cell wall production and wood hydrophobicity

**DOI:** 10.1186/s13068-025-02667-w

**Published:** 2025-06-22

**Authors:** Ashkan Amirkhosravi, Gerrit-Jan Strijkstra, Alisa Keyl, Linus Heydenreich, Cornelia Herrfurth, Ivo Feussner, Andrea Polle

**Affiliations:** 1 Forest Botany and Tree Physiology, University of Göttingen, Büsgenweg 2, 37077 Göttingen, Germany; 2 Department of Plant Biochemistry, Albrecht-von-Haller-Institute of Plant Sciences, University of Göttingen, Justus-von-Liebig-Weg 11, 37077 Göttingen, Germany; 3 Department of Plant Biochemistry, Göttingen Center for Molecular Biosciences (GZMB), University of Göttingen, Justus-von-Liebig-Weg 11, 37077 Göttingen, Germany; 4 Service Unit for Metabolomics and Lipidomics, Göttingen Center for Molecular Biosciences (GZMB), University of Göttingen, Justus von Liebig-Weg 11, 37077 Göttingen, Germany

**Keywords:** Developing xylem, Tissue-specific promoter, Lipids, Wax ester, Wood swelling, Wood density, Hydrophobicity

## Abstract

**Background:**

Many industrial applications of wood and woody biomass require harsh physicochemical pretreatments to improve the hydrophobicity and durability of the products. Environmentally friendly wood biorefineries necessitate the replacement of chemicals and energy-consuming wood processing. Here, our goal was to increase wood hydrophobicity via the ectopic expression of Jojoba (*Simmondsia chinensis*) wax ester synthase (*ScWS*) in poplar (*Populus* × *canescens*). We expressed *ScWS* under a wood-specific promoter (*DX15*), which naturally controls the expression of *FASCICLIN-like ARABINOGALACTAN PROTEIN 15* (*FLA15*) in the xylem.

**Results:**

In the *DX15::ScWS* lines, *ScWS* was highly expressed in wood but not in leaves. The transgenic lines exhibited normal photosynthesis and growth similar to the wild-type poplars. Compared with the wild-type poplars, the *DX15::ScWS* lines accumulated greater amounts of triacylglycerol in wood and a greater number of lipid droplets in ray parenchyma cells. The composition of the bark cuticle wax esters was unaffected. The wood of the *DX15::ScWS* lines showed greater water repellency and less swelling than that of the wild-type poplars. Furthermore, the *DX15::ScWS* lines had an increased expression of *FLA15* and increased cell wall deposition in fibers, resulting in increased wood density.

**Conclusions:**

Our results highlight the potential of combining the wood-specific *DX15* promoter with *ScWS* to enhance the technological properties of poplar wood. Reduced wood hydrophilicity represents a significant improvement in wood quality. In addition, our results suggest that the overexpression of the *DX15* promoter could be a promising strategy for improving lignocellulose biomass in plants. Since poplars are highly productive species that can be cultivated in short-rotation plantations, our results have high translational potential for advancing sustainable wood utilization for a wider range of applications.

**Supplementary Information:**

The online version contains supplementary material available at 10.1186/s13068-025-02667-w.

## Background

Woody biomass is a renewable resource that constitutes an important basis for biofuels and bioproducts [1, 2]. Woody biomass is produced in large amounts by fast-growing tree species cultivated in short-rotation plantations [3]. However, as a natural product, wood poses challenges to its technical and commercial applications. To overcome these difficulties, the wood of fast-growing tree species such as poplars (*Populus* sp.) has been modified by genetic engineering to increase the efficiency of lignocellulose biorefinery [4–6]. While there has been significant progress in this area of research [7], biotechnological applications to increase the suitability of wood for further added-value products are lagging behind.

To date, various physicochemical treatments are available to increase the durability and extend the service life of wood products [8]. These include, for example, chemical modification [9–12], thermal treatment [13, 14], and the application of additives or coatings [15–17]. A major obstacle to many applications is the hydrophilicity of wood, which results in uncontrolled swelling and shrinking and promotes moisture-induced degradation [18, 19]. To achieve dimensional stability of fiberboards, fibers are coated with wax during the production process [20, 21]. Furthermore, hydrophobicity and durability have been improved by the esterification of wood with long-chain fatty acids [22, 23]. Whether manipulation of the lipid content by genetic engineering can also improve the water repellency of wood remains unexplored.

In previous studies, the lipid content in the storage organs of oil seed crops was successfully increased by the overexpression of a wax ester synthase from Jojoba (*Simmondsia chinensis, ScWS*) [24]. Here, we used poplar (*Populus* × *canescens*) to investigate whether wood hydrophobicity can be increased by increasing its lipid content by the ectopic expression of *ScWS*. Since the expression of *ScWS* under the control of the constitutive *35S* promoter results in a growth trade-off [25], we decided to use a wood-specific promoter to specifically target *ScWS* expression to the xylem. This strategy may avoid off-target effects, as shown by other researchers [26–28].

The selection of the promoter is a critical step because its expression must be high, stable, and specific for the target tissue. Ko et al. [29] generated promoter-reporter constructs for several genes, which presented high expression levels in the developing xylem (DX) of poplar (‘NM6’, *Populus nigra x maximowiczii*). They showed that the promoter of *FASCICLIN-like ARABINOGALACTAN PROTEIN 15* (*FLA-like AGP15*), an ortholog of *FASCICLIN-like AGP 12* in *Arabidopsis thaliana*), targeted the expression of the reporter specifically to the DX, mature wood, and fiber bundles in the phloem but not to the leaves [29]. In the present study, we selected the homolog of the *Populus nigra* × *maximowiczii FLA-like AGP15* gene from *P. trichocarpa* (Potri.009G012200). We confirmed high expression levels of *PtFLA-like AGP15* in the DX via in silico analyses of RNA sequencing (RNAseq) data. We isolated the 5′UTR (subsequently called *DX15*) and transformed *P.* x *canescens* with the chimeric construct *DX15::ScWS*. We used wild-type (WT) and *DX15::ScWS* poplar lines to address the following questions: (1) Does the expression of *DX15*::*ScWS* affect the growth phenotype and wood anatomy of poplar; (2) does it lead to increased intracellular and cuticular lipid contents in poplar stems; and (3) does it increase the hydrophobicity of poplar wood?

## Materials and methods

### Isolation of the DX15 promoter from Populus trichocarpa

Three individual, greenhouse-grown *P*. *trichocarpa* (clone “Nisqually”) plants were used to isolate a genomic fragment flanking the 5′UTR of the Potri.009G012200 gene. In the JGI genomic database, the gene was annotated as *FLA-like AGP15* for *P. trichocarpa v3.1* and as *FLA-like AGP12 for P. trichocarpa v4.1*, http://phytozome.jgi.doe.gov). Here, we use *FLA-like AGP15* for Potri.009G012200. Genomic DNA was extracted individually from each *P. trichocarpa* plant with a DNA extraction kit (Qiagen GmbH, Hilden, Germany) according to the manufacturer’s instructions. The purity and concentration of the DNA were measured with a spectrophotometer (NanoDrop^™^ 2000, Thermo Fisher Scientific Inc., Waltham, Massachusetts, USA). After obtaining the genomic DNA, we designed gene-specific primers (Supplemental Table S1) with Geneious software (Biomatters, Ltd., Auckland, New Zealand, www.geneious.com) to amplify the *DX15* promoter sequences (*Pfu*^*®*^ proofreading polymerase, Thermo Scientific, Braunschweig, Germany). The PCR products were purified (Qiagen GmbH, Hilden, Germany) and sequenced (Microsynth Seqlab GmbH, Göttingen, Germany). The sequences obtained from the three individual plants were compared. Two sequences for the *DX15* fragment presented 100% identity, and one presented a minor deviation of 7 bp and had low sequencing quality. Resequencing confirmed the minor deviation in one plant. We selected the *DX15* sequence, which showed 100% homology between two individual plants, for further analyses. The selected promoter sequence of *DX15* was 1020 bp in length (Supplemental Figure S1a) and revealed three repeats of a TTGATAG motif instead of four in *Populus nigra* x *maximowiczii* (Supplemental Figure S1b). The *P. trichocarpa DX15* sequence deviated by an additional 11 bp from that published by Ko et al. [29] for *Populus nigra* x *maximowiczii* (Supplemental Figure S1a).

### Motif analyses in the DX15 promoter

Regulatory motifs in the 1020 bp long *DX15* promoter sequence were predicted with PlantCARE (https://bio.tools/plantcare) and PLACE (www.dna.affrc.go.jp/PLACE). Functional annotation and enrichment analysis of identified *cis*-acting elements were performed with DAVID (https://ngdc.cncb.ac.cn) and PANTHER (www.pantherdb.org) and visualized with JBrowse (https://Phytozome-next.jgi.doe.gov/jbrowse) and PlantGenie (https://plantgenie.org) (Supplemental Table S2, Supplement Figure S2).

### Generation of ScWS lines under the control of the DX15 promoter

We used gray poplar (*Populus* x *canescens*, syn. *P. tremula* x *P. alba*, clone INRA 717-1B4) for genetic transformation with wax ester synthase from *Simmondsia chinensis* (*ScWS*) under the control of the *DX15* promoter from *Populus trichocarpa* (clone “Nisqually”). Two restriction sites, *Hind*III (3′) and *Sac*I (5′), were added to the promoter sequence and ligated with the binary pK7WG Gateway vector (vector ID 4_38, VIB, Ghent, Belgium) at 16 °C for 16 h on a Thermocyler (Eppendorf, Hamburg, Germany). Specific *DX15* primers were designed with Geneious software (Biomatters) (Supplemental Table S1). The *DX15* promoter region was amplified via PCR according to the manufacturer’s instructions (Thermo Scientific, Braunschweig, Germany). The PCR products were separated by electrophoresis [30] (Supplemental Figure S3a). The amplicons were purified with the innuPREP PCRpure Kit (analytik jena AG, Jena, Germany) and then ligated upstream of the attR1 attachment site with a T4 ligase (Thermo Scientific) within the binary vector (pK7WG) according to the protocol of the manufacturer (Supplemental Figure S3b).

The Gateway cloning system (Invitrogen, Waltham, Massachusetts, USA) was used to integrate the *ScWS* into the pDONR201 vector (Thermo Fisher Scientific), and *ScWS* was inserted into the pK7WG vector according to the manufacturer’s protocol. The pK7WG-*PtDX15::ScWS* expression vector was subsequently transformed into the *Agrobacterium tumefaciens* strain GV3101 pMP90 (BacDive, Braunschweig, Germany) as described by Amirkhosravi et al. [25]. PCR colony screening was conducted to identify positive clones. For plant transformation, we used stem sections (5–19 mm long) of *P*. *x canescens* containing axillary buds from tissue culture [31]. We used the same transformation protocol described by Amirkhosravi et al. [25]) and obtained six positive transformants on selection media supplemented with kanamycin (50 mg/mL). The transformed poplar plantlets were further verified via PCR and Sanger sequencing, and two lines with the highest expression levels of *ScWS* were selected for further experiments (*DX15*::*ScWS1* and *DX15*::*ScWS2*), while other tested lines had 3- to tenfold lower expression levels.

### Plant cultivation

WT and transgenic *P*. x *canescens* plantlets (*DX15*::*ScWS1*, and *DX15*::*ScWS2*) were multiplied by micro-propagation in half-strength Murashige and Skoog (MS) media [32] in glass jars (Weck^®^ GmbH & Co. KG, Germany) as previously described [31]. The plants were cultivated under long-day conditions with a photoperiod of 16 h light/8 h dark (L18W/840 fluorescent lamps; Osram, Munich, Germany) in an air-conditioned culture room at 22 °C. Six-week-old rooted plantlets were transferred into 7 L pots with a soil and sand mixture (2 parts (*v/v*) N-type soil [Fruhstorfer Erde Type N, Hawite Gruppe GmBH, Vechta, Germany], 8 parts coarse sand (Ø 0.71–1.25 mm; Melo, Göttingen, Germany), and 2 parts fine sand [Ø 0.4–0.8 mm; Melo, Göttingen, Germany]) [31]. The plants were grown under greenhouse conditions (24 plants, experiment 1) starting in May (temperature: 23–24 °C, relative air humidity: 60–70%, ambient light supplemented with additional illumination [3071/400 HI-I; Adolf Schuch GmbH, Worms, Germany] set for a photoperiod of 16 h light/8 h dark with photosynthetically active radiation (PAR) at 150 µmol photons m^−2^ s^−1^ at plant height. The experiment was repeated in controlled climate chambers (16 plants, experiment 2) starting in August (16 h photoperiod, 23/21 °C day/night temperature, 150 μmol m^−2^ s^−1^ light and approximately 60% relative air humidity) (Supplemental Table S3). During the growth phase in the greenhouse or climate chamber, the plants were watered daily with 400 mL of distilled water per pot via a drip irrigation system (Gardena, Ulm, Germany). Furthermore, each plant was fertilized with 50 mL of Long-Ashton nutrient solution [33] every second day.

### Growth measurements

The stem diameter was measured once a week at a height of 1.5 cm above the soil surface with a digital caliper (Tchibo GmbH, Hamburg, Germany). The stem height was measured once per week from the soil surface to the apex with a folding ruler.

### Gas exchange

During the growth phase, photosynthesis, transpiration, and stomatal conductance were measured with a photosynthesis system (Flash^™^ multiphase fluorimeter LI-6800, LI-COR, Lincoln, USA). Measurements were conducted between 10 a.m. and 2 p.m. with a light intensity of 800 µmol photons m^−2^ s^−1^ of PAR, a 25–27 °C leaf temperature, and a CO_2_ concentration of 400 µmol mol⁻^1^ on 5 individual plants per line. We used a fully developed, light-exposed top leaf, usually leaf #7 from the stem apex, for gas exchange measurements.

### Harvest and sampling procedures

The plants were harvested after 100 days of greenhouse cultivation. The plants in the climate chambers were cultivated for 90 days and then harvested. Each part (stem, leaf, and root) was harvested separately. All the fractions were immediately weighed to determine the total fresh biomass of each tissue. Aliquots of each tissue were collected, weighed fresh and dry after 1 week at 60 °C. The dry weight per tissue (g) was calculated as follows:

$$\frac{{{\text{Dry weight of aliquot }}\left( {\text{g}} \right) \times {\text{ Total tissue biomass fresh (g)}}}}{{\text{Fresh weight of aliquot (g)}}}$$

During harvest, aliquots of fresh leaves were shock-frozen in liquid nitrogen and stored at − 80 °C. Furthermore, three fresh leaves from the bottom, middle, and top of the plants were weighed and photographed with a digital camera (Sony α6400, Tokyo, Japan). The leaf area was determined via ImageJ software (imagej.net/ImageJ, [34]. The whole plant leaf area was calculated as follows:

$$ \frac{{{\text{Total\, plant\, leaf\, fresh\, weight\, }}\left( {\text{g}} \right) \times {\text{ scanned\, leaf\, area}}\,({\text {cm}}^{2})}}{{\text{Fresh\, weight\, of\, scanned\, leaves\, (g)}}} $$

During harvest, several fresh stem segments were collected at defined positions in the stem (Supplemental Figure S4a). The stem sections used for anatomical analysis and histochemistry were stored in FAE solution (1:1:18, [*v/v/v*]; 36% [*v/v*] formaldehyde:96% [*v/v*] acetic acid:70% [*v/v*] ethanol) (Supplemental Figure S4b). For cuticular wax analysis, fresh bark samples were taken from the positions indicated in Supplemental Figure S4c and debarked wood was stored at − 80 °C for lipid analysis. Stem samples for wood properties (position indicated in Supplemental Figure S4d) were immediately used or dried as required. For molecular analyses (Supplemental Figure S4e) samples were dissected into DX, wood, and bark [35] and stored at − 80 °C after shock freezing in liquid nitrogen.

### RNA extraction and quantitative real-time (qRT)-PCR

The frozen leaf, wood, bark, and DX samples were milled to a fine powder in a precooled ball mill (Retsch, Hann, Germany). A total of 150 mg of frozen powder was subsequently used for total RNA extraction with the hexadecyltrimethylammonium bromide (CTAB) method [36]. The quantity and quality of total RNA were determined with a spectrophotometer (NanoDrop^™^ 2000, Thermo Fisher Scientific Inc., Waltham, Massachusetts, USA). Genomic DNA was eliminated according to the manufacturer’s instructions with a Turbo DNA-free kit (Turbo DNA-free kit, Ambion, Austin, TX). The integrity of the purified RNA (2.5 μg) was confirmed via agarose gel electrophoresis [37]. Total RNA (5 µg) served as the starting material for double-stranded cDNA synthesis, and the Oligo(dT)18 primer and the RevertAid H Minus First Strand cDNA Synthesis Kit (Thermo Fisher Scientific Inc., Waltham, Massachusetts, USA) were used according to the manufacturer’s protocol. The primers used for qRT‒PCR were designed via Perl Primer software version 1.1.20 [38]. The primer sequences are shown in Supplemental Table S1. qRT‒PCR assays were conducted on an optical 96-well plate via the Jena Quantitative Real-time PCR tower system (qTOWER3 G touch, Analytik Jena, Jena, Germany). All qRT‒PCRs were conducted with the following conditions: initial denaturation at 95 °C for 2 min; 45 cycles of denaturation at 95 °C for 10 s and annealing/extension at 55 °C for 20 s; and melting curve analysis with measurements between 72 °C and 95 °C. Relative gene expression was calculated with the 2^−ΔΔCt^ method [39] and normalized against two reference genes, *PtrPPR_2* (Potri.012G141400) and *PtrRpp14* (Potri.015G001600).

### Determination of wood density and water uptake

We used stem sections (Supplemental Figure S4d) 10 cm in length for the determination of wood density and water uptake. Fresh samples (debarked) were weighed and submersed in a graduated cylinder (Megro, Wesel, Germany) with double-deionized water; the amount of displaced water was taken as the wood volume. The wood density (g cm^−3^) of the samples was calculated as follows:

$$\rho = \frac{mass \left( g \right)}{{volume \left( {cm^{3}} \right)}}$$

Stem segments 10 cm in length (Supplemental Figure S4d) were debarked, freeze-dried (Piatkowski Forschungsgeräte-Vertrieb, München, Germany) for five days and weighed instantly (m_d_). The samples were placed in sealed reaction vessels (50 mL Falcon^™^; Fisher Scientific GmbH, Schwerte, Germany) filled with 40 mL of tap water. After 24 h at room temperature, the stem weight was again measured (m_w_) and the relative wood water uptake was calculated [40] as follows:

$${\text{Relative\, wood\, water\, uptake\,}} \Delta 24{\text{h}} = \frac{{\left( {{\text{m}}_{{\left( {\text{w}} \right)}} - {\text{m}}_{{\left( {\text{d}} \right)}} } \right)}}{{{\text{m}}_{{\left( {\text{d}} \right)}} }}$$

### Water contact angle measurements

Dry, debarked stem segments were used. Water droplets of 10 µL were carefully placed at three different positions on the wood surface of each sample, and images of the droplets were captured with a digital camera equipped with a 135 mm lens (Sony α6400, Tokyo, Japan). The images were analyzed with ImageJ software, which was equipped with the contact angle plugin (https://bigwww.epfl.ch/demo/dropanalysis/#soft). To determine the contact angle, the cursor was placed at the point of contact between the water droplet and the wood surface to initiate angle measurement [41]. The measurements were repeated for each droplet on a sample and averaged to obtain one mean value per sample. Four to five individual plants were analyzed per poplar line.

### Wood anatomy

We prepared cross-sections from stem segments (Supplemental Figure S4a) according to Euring et al. [42] with the following modifications: wood segments, which had been stored in FAE, were sectioned at − 30 °C in a cryomicrotome (Cryostat CM 3050S, Leica Biosystems, Nussloch GmbH, Nussloch, Germany) equipped with a low-profile microtome blade (low-profile disposable blades 819; Leica Biosystems). Cross-sections of 20 µm thickness were cut and kept in tap water until further processing. The sections were stained with 0.05% (*w/v*) toluidine blue (Merck KGaA, Darmstadt, Germany) solution (pH 7.0) [43] for 7 min, washed twice in double-deionized water, mounted in 50% (*v/v*) glycerol, and immediately viewed under a light microscope (Zeiss Axioplan; Carl Zeiss AG, Oberkochen, Germany). Images were acquired with a digital camera (AxioCam MR3 microscope camera; Carl Zeiss AG, Oberkochen, Germany) at 100- and 400-fold magnifications. The digital images were analyzed with ImageJ software (http://rsbweb.nih.gov/ij/; NIH) to determine the frequencies and lumina of vessels, fibers and ray cells, as well as the cell wall thickness of fibers and vessels, as described previously [42]. The area of an individual ray was determined by measuring its length from the pith to the cambium and its width at distinct positions, such as the start of the mature xylem, at the middle between the pith and cambium and close to the cambium. This procedure was repeated for three rays per slide, and the mean ray area of the sample was calculated. The total cell wall area was determined in a selected area in secondary wood by subtracting the fiber area, vessel area and ray area from the selected area.

### Surface wax analysis

The surface waxes were extracted from the bark of the *DX15*::*Sc*WS lines and the WT. Bark samples were collected from the lower stem region, 5 cm above the soil surface (Supplemental Figure S4d). The bark sections were cut into strips 50 mm in length × 5 mm in width. The fresh bark strips were submerged for 30 s in 5 mL of chloroform with 2.5 µg of *n*-tetracosane and further processed as previously described [44]. The derivatized samples were separated via gas chromatography and mass spectrometry as described by Amirkhosravi et al. [25]. We detected minute amounts of unusual even-numbered alkanes (C26, C28) and odd-numbered fatty acids (C25, C27). These were likely contaminants and were therefore not included in the analyses.

### Lipid content of wood

The analysis of triacylglycerol, wax esters and total fatty acids was performed as described by Iven et al. [45] with some modifications. Triacylglycerol and wax esters were analyzed from 100 mg of freeze-dried wood powder with 40 µg of tripentadecanoate and 20 µg of heptadecanoyl heptadecanoate for quantification after thin-layer chromatography and acidic methanolysis by gas chromatography‒flame ionization detection (GC‒FID). The total fatty acid composition and content were determined in 10 mg of dried wood powder with 20 µg of tripentadecanoate for quantification after acidic methanolysis.

### Histochemistry of lipids in wood

We stained lipid droplets in wood cells following the protocol of DiDonato and Brasaemle [46] as follows: stem samples stored at − 80 °C (Supplemental Figure S4d) were defrosted in double-deionized water, cut into smaller pieces, and transferred into a 1.5 mL safe lock reaction tube (SARSTEDT AG & Co. KG, Nümbrecht, Germany) containing 1000 µL paraformaldehyde (20 mL 5% (*v/v*) potassium dichromate, 5 mL 37% (*v/v*) formaldehyde, and 1.25 mL 100% (*v/v*) citric acid). The samples were exposed to vacuum for 5 min at − 600 bar to remove air bubbles and improve fixative penetration. Thereafter, the samples were incubated for 20 h at room temperature in the dark. The fixed samples were then removed from the fixative and washed twice with distilled water. The samples were frozen in a small aluminum box at − 20 °C. Cross-sections of 30 µm thickness were prepared in a cutting chamber at − 30 °C with a low-profile microtome blade (low-profile disposable blades 819; Leica Biosystems Nussloch GmbH, Nussloch, Germany). The cross sections were stained with LipidSpot^™^ 488 lipid droplet stain (Biotium Inc., Fremont, California, USA) according to the manufacturer’s protocol with minor modifications: 1 µL of LipidSpot^™^ 488 lipid droplet stain was mixed with 1000 µL of phosphate-buffered saline (PBS). The cross sections were subsequently incubated in the staining solution at 37 °C for 10 min in the dark. Eventually, the cross sections were embedded with 10 µL of ROTI^®^Mount FluorCare PI (Carl Roth GmbH + Co. KG, Karlsruhe, Germany) on slides. Fluorescence images were obtained via a confocal laser scanning microscope (Leica TCS SP8; Leica Microsystems GmbH, Wetzlar, Germany) with an excitation wavelength of 480–490 nm and an emission wavelength of 580–620 nm. Digital images were recorded at 10 × and 40 × magnification. The number of lipid droplets was counted in three randomly selected ray parenchyma files along a 200 µm-long line in the middle of the ray per cross section. All the droplets either on the line or connected to the line were counted. Four different individuals of each poplar line were analyzed.

### Analysis of FLA expression levels in RNAseq datasets

We retrieved the transcript abundances for Potri.009G012200 in openly available RNAseq datasets for *P*. x *canescens:* DX and mature wood (European Nucleotide Archive: E-MTAB-7288, [47]) and leaves (E-MTAB-12654, [48]. We used RNAseq datasets for three *P. nigra* genotypes (“Italy”, “France”, and “Spain”): DX (NCBI short-read archive under SRP number SRP095832) and leaves and fine roots (NCBI SRP101711) [49]. We show the means for count data (n = 3 or 4 biological replicates per tissue ± SE).

### Statistical analyses

R version 4.2.2 software [50] and Origin 2020 software (OriginLab Corporation, Northampton, Massachusetts, USA) were used for statistical analysis. We tested the normal distribution of the data and then used one-way analysis of variance (ANOVA) to test for significant effects among the lines (WT, *DX15*::*ScWS1*, *DX15*::*ScWS2*) at *p* < 0.05. The post hoc Tukey test was applied to determine significant differences between means at *p* < 0.05. For all measurements and statistical analyses, the number of individual biological replicates and the statistical tests are indicated in the figure legends. The data are shown as the means (± SE).

## Results

### The DX15 promoter is specific to wood and shows conserved motifs across different species

**Fig. 1:**
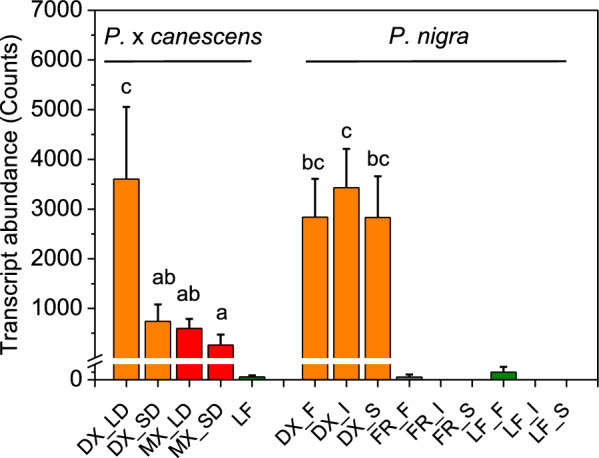
Transcript abundances of *FLA-like AGP15* in different tissues of *P.* x *canescens* and *P*. *nigra.* The transcript abundances of *FLA-like AGP15* (the best match to Potri.009G012200) were extracted from RNAseq datasets for *P*. x *canescens* (clone INRA 717-1B4) and three genotypes of *P. nigra* (F = France, I = Italy, S = Spain). The following tissues were included: wood under long-day (LD) and short-day conditions (SD) separated into developing xylem (DX, orange), mature xylem (MX, red), leaves (LF, green) and fine roots (FR, gray). Different letters indicate significant differences among the woody tissues. The data are presented as means for normalized count data (n = 3 or 4 biological replicates per tissue and genotype, ± SE)

We observed that *FLA-like AGP15*, the gene controlled by *DX15*, is highly expressed in DX and is expressed at intermediate levels in mature wood across different poplar genotypes (Fig. 1). Leaves and fine roots presented either no or marginal transcript abundances of *FLA-like AGP15* (Fig. 1). We further demonstrated that *FLA-like AGP*15 was responsive to the light phase, since the transcript abundances of *FLA-like AGP*15 from short-day (11 h light) poplars were significantly lower than those from long-day (16 h light)-grown poplars (Fig. 1).

We isolated the promoter region (1020 bp upstream of the start codon) of the putative *FLA*-like *AGP15* from *P*. *trichocarpa* and named the fragment *DX15*, in accordance with the annotation proposed by Ko et al. [29]. We detected 99% nucleotide homology of the *DX15* promoter region of *P*. *trichocarpa* with that of *P*. *nigra* × *maximowiczii* (Supplemental Figure S1). Comparative in silico analysis of the *DX15* region with that of several other species revealed fifteen CAAT boxes in the sequence ranging from − 788 to − 61 bp. CAAT boxes are known for modulating tissue-specific promoter activity [51]. The promoter region contained numerous DNA-binding domains (DOFs = DNA one-finger transcription factors) (Supplemental Figure S2, Supplemental Table S2), which have regulatory functions in secondary cell wall formation [51]. Furthermore, we identified conserved *cis*-acting motifs involved in light regulation, abiotic stress, and hormone responses (Supplemental Figure S2, Supplemental Table S2). The presence of these conserved *cis*-acting elements in poplar as well as other plant species supports their evolutionary conservation.

### Transgenic poplars show high DX15::ScWS expression in wood and normal growth phenotypes

**Fig. 2:**
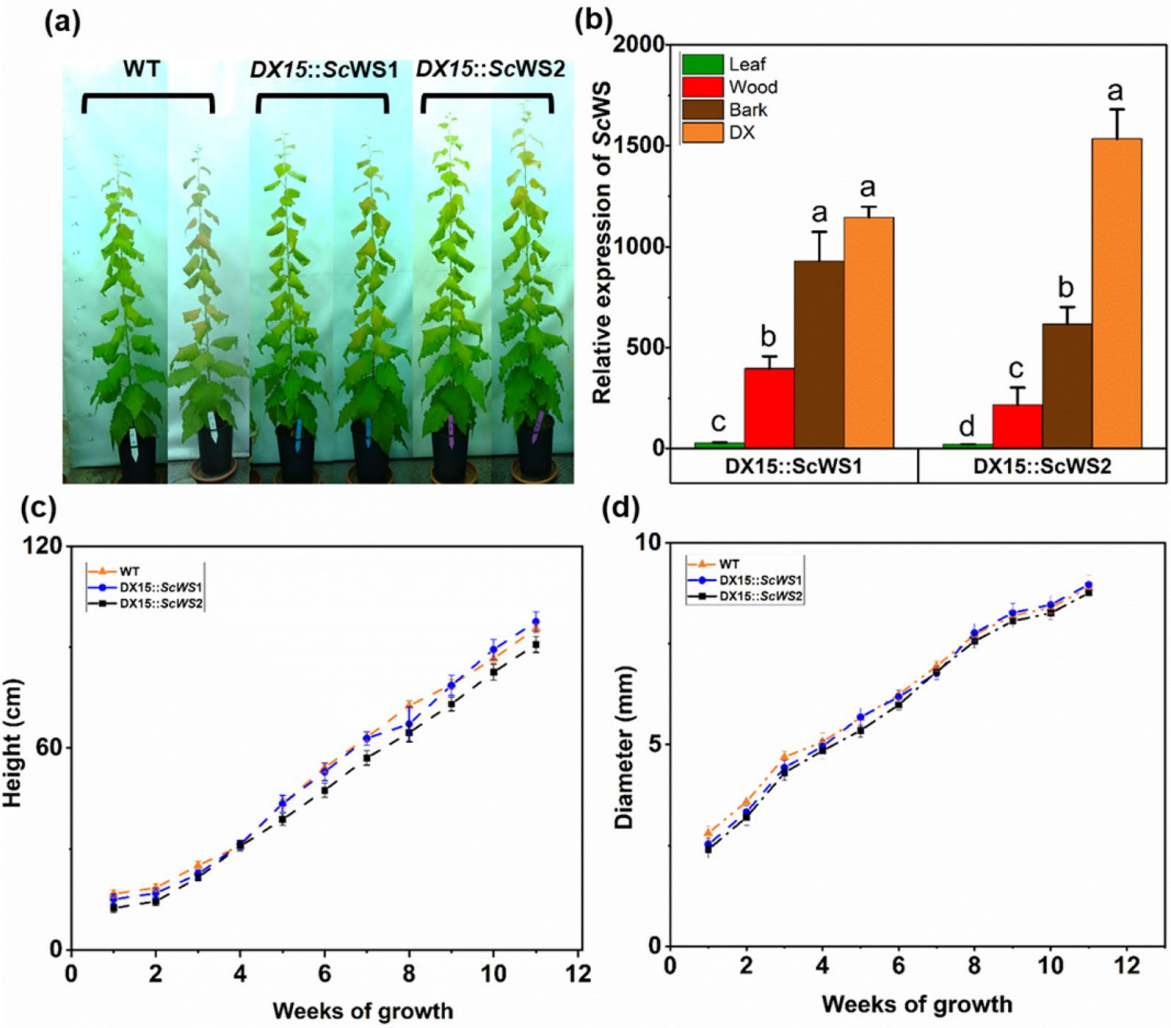
Phenotype (**a**), relative transcript abundances of *ScWS* (**b**), stem height (**c**) and diameter growth (**d**) of *P.* x *canescens* wild-type (WT) and two *DX15*::*ScWS* lines. The plants were grown for 100 days under semicontrolled greenhouse conditions with supplementary light (16 h day length). Height and diameter were measured weekly (means for n = 8 per line, ± SE). There were no significant differences among the lines at p < 0.05. The relative transcript levels of wax ester synthase (*Sc*WS) were determined in the *DX15*::*Sc*WS poplar lines in leaves, bark, mature wood and developing xylem and normalized to housekeeping gene expression levels. The data are presented as the means (n = 4, ± SE), and different letters indicate significant differences among the tissues at p ≤ 0.05 (one-way ANOVA, post hoc Tukey test)

We expressed *ScWS* under the control of the *DX15* promoter in *P*. x *canescens*. Among six poplar lines expressing *ScWS*, we selected *DX15*::*ScWS1* and *DX15*::*ScWS2* for further analyses. The transgenic plants presented a normal phenotypic appearance (Fig. 2a). *DX15*::*ScWS1* and *DX15::ScWS2* exhibited high expression levels of *ScWS* in stem tissues (Fig. 2b), indicating that the chimeric construct was functional in *P*. x *canescens*. The highest relative transcript abundances for *ScWS* were present in DX, followed by bark and mature wood (Fig. 2b). The transformed plants exhibited very low expression levels of *ScWS* in leaves under the control of *DX15* (Fig. 2b). However, the transcript abundances of *ScWS* were more than 1200-fold higher in DX than in leaves (Fig. 2b).

The WT, *DX15::ScWS1*, and *DX15::ScWS2* plants had similar rates of transpiration (mean ± SE across all lines: 5.07 ± 0.53 mmol H_2_O m^−2^ s^−1^), stomatal conductance (0.25 ± 0.018 mol m^−2^ s^−1^), and photosynthesis (6.6 ± 0.483 µmol CO_2_ m^−2^ s^−1^) (Table 1). This was confirmed in an independent experiment conducted in controlled climate chambers (experiment 2, Supplemental Table S3). The stomatal length and density of the WT leaves were similar to those of the *DX15::ScWS* lines (Supplemental Figure S5a–h).

**Table 1:** Photosynthesis, stomatal conductance and transpiration of wild-type (WT) and *DX15::ScWS*-expressing poplar lines

	WT	*DX15::ScWS1*	*DX15::ScWS2*	*p*_*line*_
Photosynthesis (µmol m^−2^ s^−1^)	6.79 ± 0.38	6.94 ± 0.76	6.26 ± 0.23	0.12
Stomatal conductance (mol m^−2^ s^−1^)	0.25 ± 0.04	0.25 ± 0.04	0.23 ± 0.04	0.44
Transpiration (mmol m^−2^ s^−1^)	5.57 ± 0.46	4.87 ± 0.77	5.28 ± 1.18	0.51

Gas exchange measurements were conducted once a week during the 100-day experimental period from 10 am to 3 pm. The average air temperature and ambient air humidity during the measurement were 23.5 ± 0.3 °C and 58% ± 2%, respectively. The photon flux density of the photosynthetically active radiation was set to 800 μmol photons m^−2^ s^−1^ at the leaf level. The data are presented as the means (n = 5 plants per line, ± SE) (General linear model with time as random factor and line as a fixed factor, ANOVA)

We did not detect any significant differences in height or diameter growth between the transgenic lines (*DX15::ScWS1* and *DX15::ScWS2*) and the WT during the 3-month cultivation period (Fig. 2c, d). The mean growth rates were 8.2 mm day^−1^ for height increments and 52 µm day^−1^ for diameter increments across the WT and transgenic lines. At harvest, the transgenic *DX15::ScWS* poplars had significantly greater whole-plant leaf areas than the WT (Table 2). Furthermore, the *DX15::ScWS* lines produced slightly greater leaf and stem biomass than did the WT under greenhouse conditions (Table 2), whereas no differences were detected in the climate chamber experiment with these lines (Supplemental Table S3). Overall, growth and physiology were either similar to or slightly greater in the *DX15::ScWS* lines than in the WT.

**Table 2:** Morphology and biomass of wild-type (WT) and *DX15::ScWS*-expressing poplar lines

Parameter	WT	*DX15::ScWS1*	*DX15::ScWS2*	*p*_line_
Number of leaves	43.8 (0.5) a	42.5 (1.3) a	43.4 (0.7) a	0.570
Stem height (cm)	92.7 (1.3) a	91.9 (1.9) a	87.9 (1.4) a	0.078
Stem diameter (mm)	7.31 (0.12) a	7.29 (0.17) a	7.18 (0.13) a	0.790
Leaf size (cm^2^ leaf^−1^)	1086 (205) a	1575 (757) a	1025 (226) a	0.251
WP* leaf area (cm^2^ plant^−1^)	3259 (615) a	4727 (227) b	4867 (461) b	0.041
Biomass of leaves (g plant^−1^)	9.7 (0.4) a	11.0 (0.8) b	10.5 (0.4) ab	0.050
Biomass of stem (g plant^−1^)	3.3 (0.2) a	5.0 (0.4) b	4.2 (0.3) ab	< 0.001
Biomass of roots (g plant^−1^)	6.3 (0.6) a	8.6 (0.7) a	8.0 (0.8) a	0.066
WP biomass (g plant^−1^)	19.3 (0.4) a	24.6 (0.6) b	22.7 (0.5) b	0.003
Root-to-shoot ratio	0.51 (0.4) a	0.55 (0.4) a	0.54 (0.03) a	0.802

*WP = whole plant

We used 100-day-old poplar plants for the measurements. The data are presented as the means (n = 5 per line, ± SE). Different letters indicate significant differences among the lines at P ≤ 0.05 (one-way ANOVA, post hoc Tukey test)

### DX15::ScWS lines produce denser wood

**Fig. 3:**
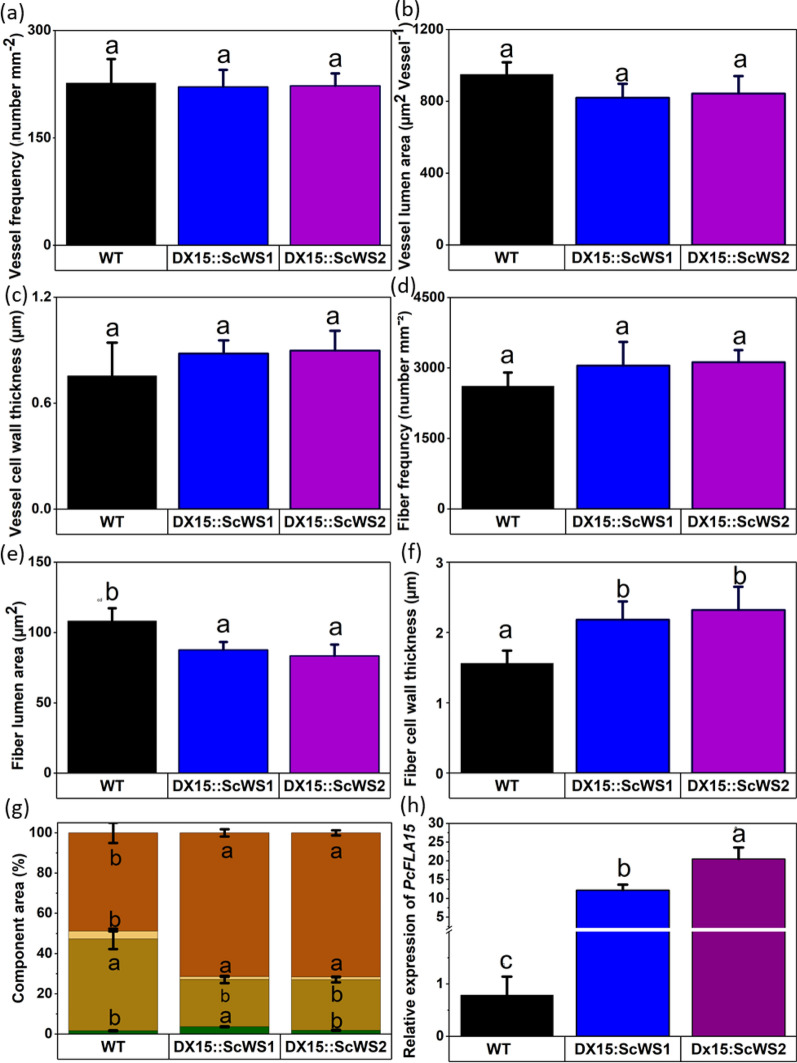
Wood anatomy and expression of *FLA-like AGP15* in wild-type (WT) and *DX15*::*ScWS*-expressing poplar lines. The anatomical characteristics of wood (Supplemental Figure S6) from approximately 3-month-old poplars were determined in cross sections. **a** Vessel frequency (number mm^−2^), **b** vessel lumen area (µm^2^ vessel^−1^), **c** vessel cell wall thickness (µm), **d** fiber frequency (number mm^−2^), **e** fiber lumen area (µm^2^ fiber^−1^), **f** fiber cell wall thickness (µm), **g** proportion of different cell types per area unit (orange = cell wall area, yellow = vessel lumina, brown = fiber lumina, green = ray parenchyma), **h** relative transcript abundance of *FLA-like AGP15* in the developing xylem of the WT and *DX15*::*Sc*WS poplar lines. The expression levels were normalized to the housekeeping gene expression levels. The data represent the means (n = 6 individual plants per line and treatment ± SE). The data were analyzed via ANOVA and the post hoc Tukey test. Different letters indicate significant differences among the lines at p ≤ 0.05

We asked whether the expression of *DX15::ScWS* altered wood anatomy compared with that of the WT. Compared with WT, *DX15::ScWS* expression did not influence vessel lumina, vessel frequency, or vessel cell wall thickness (Fig. 3a–c; Supplemental Figure S6). The fiber frequencies also did not differ between the *DX15::ScWS* lines and the WT (Fig. 3d). However, we detected smaller fiber lumina (Fig. 3e) and thicker fiber cell walls (Fig. 3f) in the wood of the *DX15*::*ScWS* lines than in that of the WT. The ray width was also greater in the *DX15::ScWS* lines than in the WT (*p* = 0.004, Supplemental Figure S6e).

We further compared the fractions of lumina, ray area, and cell wall area per cross-sectional area (Fig. 3g, Supplemental Figure S6). Overall, we detected a greater proportion of cell walls and a lower proportion of cell lumina in the wood of the *DX15::ScWS* lines than in that of the WT (Fig. 3g). Higher cell wall production in the *DX15::ScWS* lines was unexpected. Therefore, we studied the transcript abundance of *FLA-like AGP15*, i.e., the structural gene, which is controlled by the *DX15* promoter. We found drastic overexpression of *FLA-like AGP15* in the *DX15::ScWS* lines compared with the WT (Fig. 3h).

**Fig. 4:**
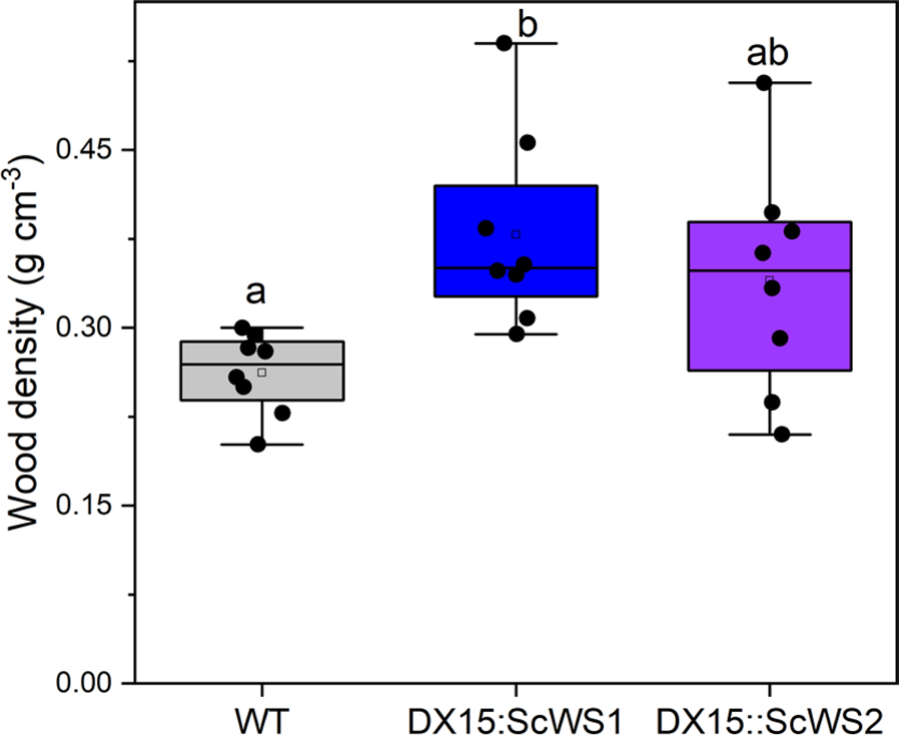
Wood density of wild-type (WT) and *DX15*::*ScWS*-expressing poplar lines. The data are presented as the means (n = 8 individual plants per line, ± SE). Different letters indicate significant differences among the treatments and lines at P ≤ 0.05 (one-way ANOVA, post hoc Tukey test)

Wood densities were greater in the *DX15::ScWS* lines than in the WT (Fig. 4). Compared with the WT line, the *DX15::ScWS1* line showed a significant increase (+ 50%, *p* = 0.01) in wood density, whereas the *DX15::ScWS2* line presented an intermediate increase (+ 30%) (Fig. 4). Greater wood densities in *DX15::ScWS1* plants than in *DX15::ScWS2* plants were also confirmed in experiment 2 (Supplemental Table S3).

### Expression of DX15::ScWS results in increased triacylglycerol content in wood but does not affect the cuticular wax composition of the bark

**Fig. 5:**
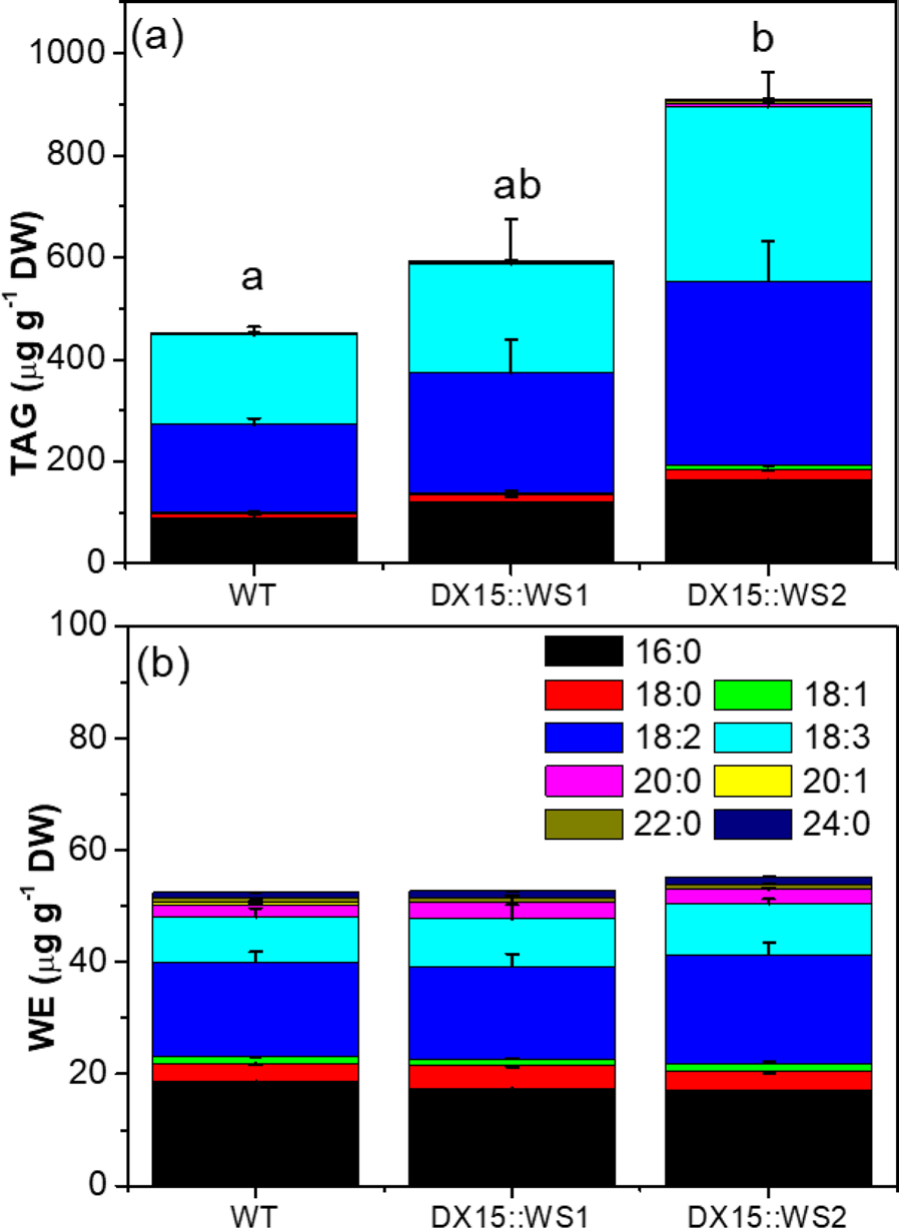
Composition and quantities of triacylglycerol (**a**) and wax esters (**b**) in the wood of *P*. x *canescens* lines. Cn:x indicates the number of aliphatic C atoms and double bonds (**a**) in the fraction of triacylglycerol (TAG) and (**b**) in the fraction of potential wax esters (WE). Significant differences at p < 0.05 are indicated by different letters for TAGs (ANOVA, Tukey HSD test). The components in the WE fraction did not significantly differ

Next, we measured the total fatty acid, triacylglycerol, and wax ester contents in the wood. The resulting fractions were composed of fatty acids with carbon chain lengths from C16 to C24 [C16:0, C18:0, C18:1 (9*Z*), C18:2 (9*Z*, 12*Z*), C18:3 (9*Z*, 12*Z*, 15*Z*), C20:0, C20:1 (11*Z*), C22:0, C24:0] (Fig. 5). No significant differences in total fatty acid content (sum of all compounds: 4.4 ± 0.2 µg g^−1^ dry weight) were detected between the WT and transgenic lines. However, the fraction of fatty acids detected in triacylglycerol was significantly greater in *DX15::ScWS2* than in WT (*p* = 0.04), whereas the triacylglycerol content in *DX15::ScWS1* was intermediate between that in WT and *DX15::ScWS2* (Fig. 5a). The wax ester fraction contained only low amounts of fatty acids (sum of all the compounds < 0.05 µg g^−1^ dry weight, Fig. 5b). Neither wax esters (C36–C48) nor other typical compounds for waxes, i.e., aldehydes (C26, C28), alkenes and alkanes (C25–C29), or primary alcohols (C22–C30), were detected (Fig. 5b). Since wax esters are the product of primary alcohols and fatty acids [52], the lack of primary alcohols in wood may have prevented wax ester formation by the innate WS or the introduced ScWS.

In contrast to wood, the cuticular wax composition of poplar bark consisted of very long-chain fatty acids (C20–C28), aldehydes (C26, C28), odd-numbered alkenes and alkanes (C25, C27, C29), primary alcohols (C22–C30), and wax esters (C36–C48) (Supplemental Figure S7). The amount of each substance class, the total wax load, and the carbon chain length distribution pattern of the bark waxes did not significantly differ between the WT and the lines *DX15::ScWS1* and *DX15::ScWS2* (Supplemental Fig. S7) and was similar to that previously reported for WT poplar leaves [25, 53].

**Fig. 6:**
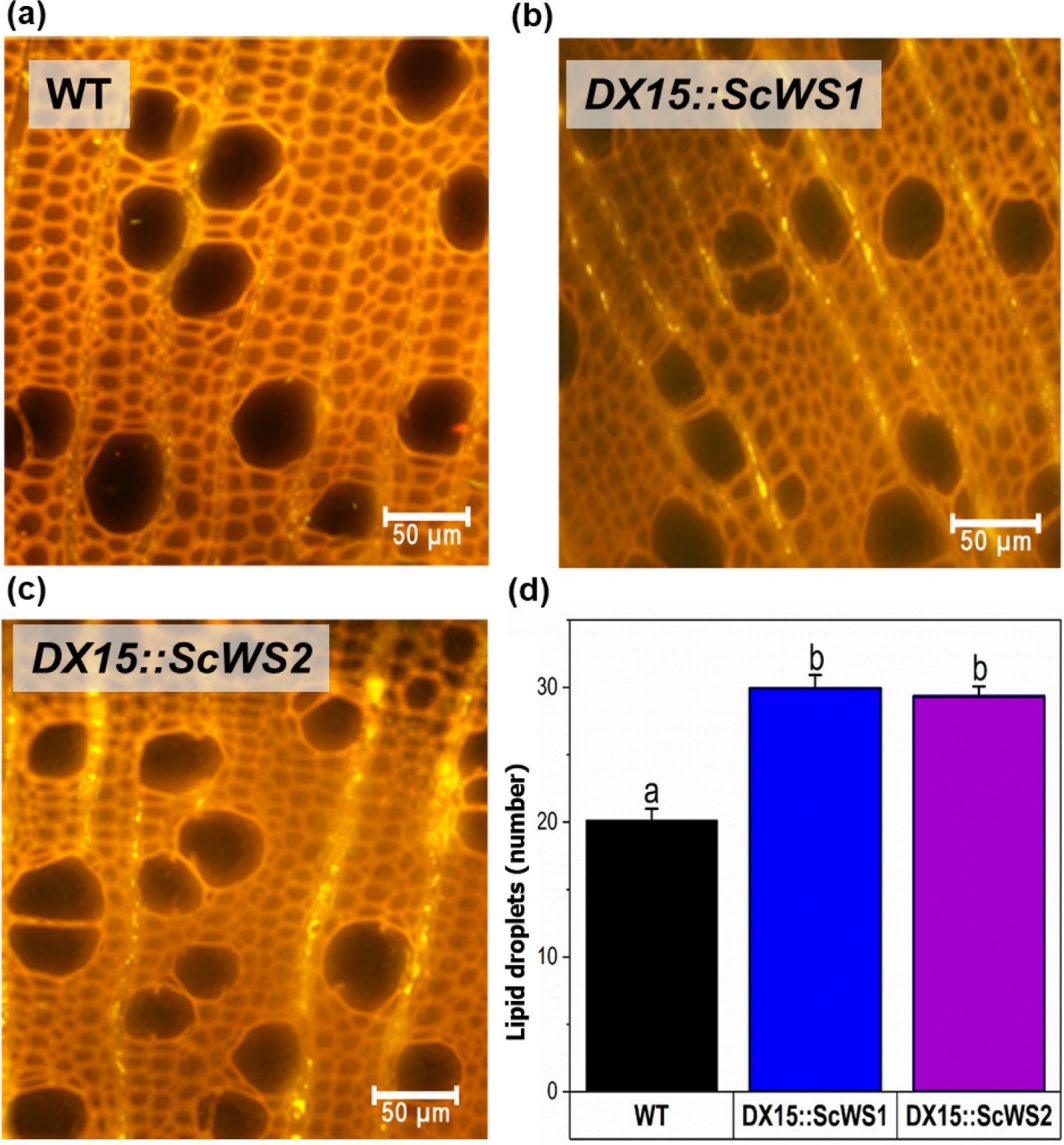
Localization and quantification of lipid droplets in wood of wild-type (WT) and *DX15::ScWS*-expressing poplar lines. Wood cross sections of *P*. x *canescens* WT (**a**), *DX15*::*Sc*WS1 (**b**) and *DX15*::*Sc*WS2 (**c**) were stained with lipophilic Spot Dye and imaged via fluorescence microscopy. Lipid droplets (yellow spots) were detected in ray parenchyma cells and counted along 200-µm-long transects in three different ray parenchyma files per cross section. The data represent the means (n = 4 individual plants per poplar line ± SE). Different letters indicate significant differences among the lines at p ≤ 0.05 for log-transformed data (ANOVA and post hoc Tukey test)

Imaging of wood cross sections after staining with a fluorescent lipophilic dye revealed the presence of lipid droplets in the ray parenchyma cells of poplar wood (Fig. 6a–c). In the WT, lipid droplets occurred mainly in the vicinity of vessels (Fig. 6a), whereas in the *DX15*::*ScWS1* and *DX15*::ScWS2 plants, they were spread throughout the whole file of ray parenchyma cells (Fig. 6b, c). The number of lipid droplets (per ray) was approximately 1.5-fold greater in the *DX15*::*Sc*WS lines than in the WT (Fig. 6d).

### Expression of DX15::ScWS enhances wood hydrophobicity

**Fig. 7:**
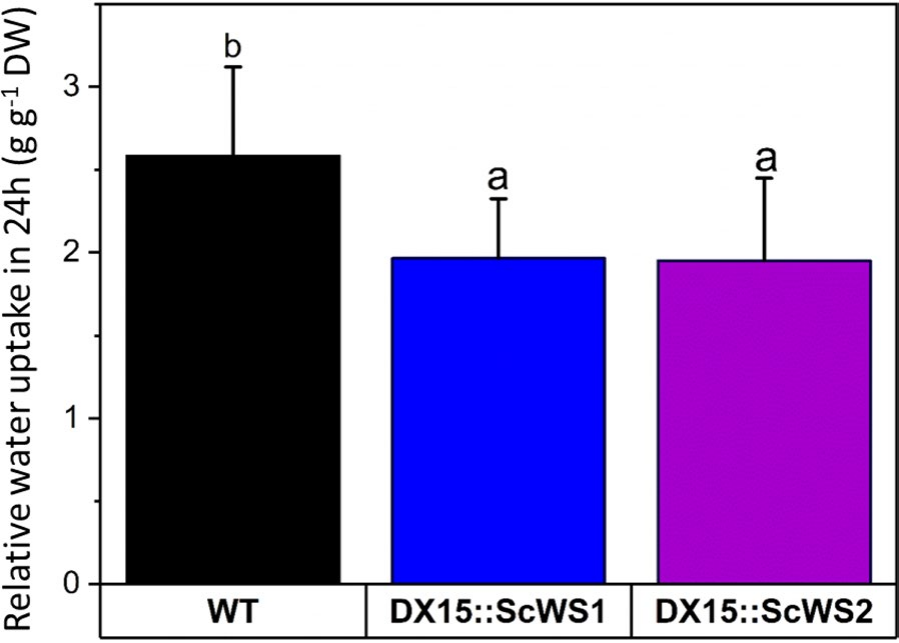
Water uptake of wood from wild-type (WT) and *DX15*::*ScWS*-expressing poplar lines. Wood water uptake was determined as the difference in weight of dry stem wood sections before and after being submerged for 24 h in water. The data are expressed relative to the initial dry weight. The data are presented as the means (n = 16 individual plants per line, ± SE). Different letters indicate significant differences among the lines at p ≤ 0.05 for log-transformed data (ANOVA, post hoc Tukey test)

To evaluate the effect of lipid accumulation in the *DX15::ScWS* lines on the wettability of the wood, we determined the relative wood water uptake of freeze-dried debarked stem sections. The relative water absorption by stems of the *DX15::ScWS* lines was 25% lower than that of the WT (*p* = 0.020) (Fig. 7).

**Fig. 8:**
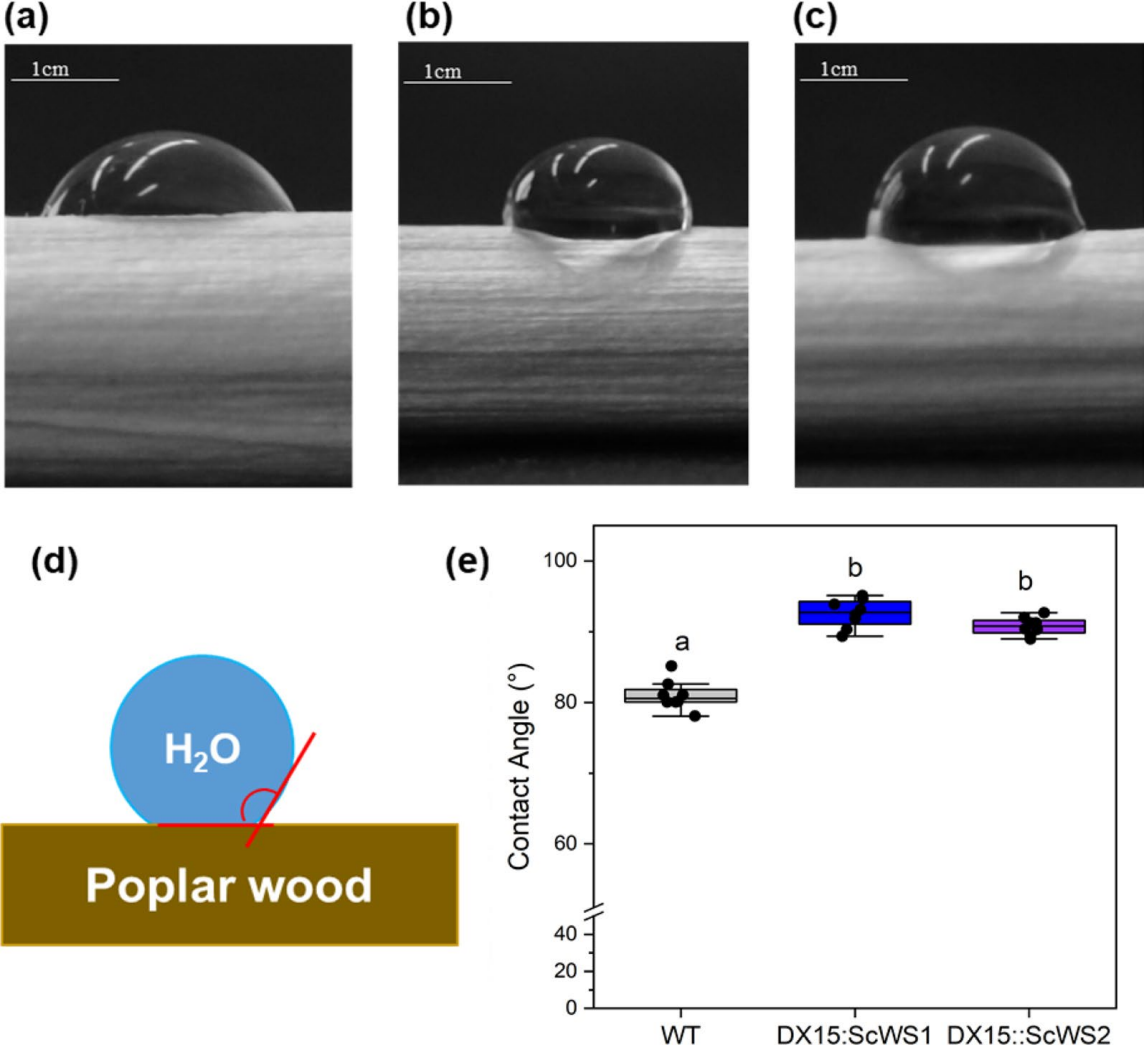
Hydrophobicity of wood surfaces from wild-type (WT) and *DX15*::*ScWS*-expressing poplar lines. Illustration of water drops (10 µL) placed on debarked dry wood surfaces of WT (**a**), *DX15::Sc*WS1 (**b**), and *DX15::Sc*WS2 (**c**) poplar lines, scheme for analysis of water contact angles (**d**) and means of water contact angles (**e**). Images were taken of three water drops per wood sample. The data are presented as the means (n = 8 plants per line, ± SE) (ANOVA, Tukey post hoc test)

We further investigated the water repellency of the wood surfaces (Fig. 8). Water droplets placed on debarked wood exhibited a more hemispheric shape on the wood surfaces of the *DX15::ScWS* lines than on those of the WT (Fig. 8a–c). The water contact angle reflects the degree of hydrophobicity, with higher angles indicating greater water repellency, as illustrated in the scheme in Fig. 8d. Compared with that of the WT, the water contact angle on the wood surfaces of the *DX15::ScWS* lines was significantly greater, with a contact angle of almost 90° (Fig. 8e).

## Discussion

### Expression of ScWS under the control of DX15 results in increased accumulation of lipid droplets in poplar stems

In the present study, we present an innovative approach to increase the hydrophobicity of wood. We successfully expressed wax ester synthase from Jojoba in poplar wood, resulting in increased lipid droplet numbers and the accumulation of triacylglycerol. Previous studies targeted *ScWS* to seeds of oil seed crops [45, 54–56]; this approach resulted in increased amounts of wax esters in the storage organs but had negative effects on seed germination [24]. In the leaves of poplar plants overexpressing *ScWS* under the ubiquitous *35S* promoter, increased lipid droplets were also detected, but the transgenic plants showed decreased stomatal conductance, aberrant lipid accumulation in guard cells and a reduction in their photosynthetic capacity [25]. In the present study, there was no indication that specific targeting of *ScWS* expression to developing wood had negative effects on the photosynthetic performance of the transgenic poplars. Poplar growth depends on water availability and leaf area formation [57]. The transpiration rates of the transgenic poplars were similar to those of the WT and the leaf areas in most cases greater than those of the WT, indicating that the accumulation of lipids in the stem neither affected water transport through the hydraulic system of the plants nor had negative impacts on height or radial stem growth. Thus, our results emphasize the utility of the tissue-specific expression of candidate genes for wood improvement.

In a previous study, we observed that overexpression of *35S::ScWS* resulted in a moderate decrease in the cuticular wax load [25]. We speculated that increased intracellular lipid production diverted precursors for cuticular waxes to intracellular cellular sinks [25]. In the present study, *DX15::ScWS* expression was high in the bark but did not affect the amounts or composition of bark cuticular waxes, suggesting that there was no trade-off between cuticle formation and intracellular lipid deposition.

In natural poplar wood, storage lipids are minor components [58, 59]. They are deposited as triacylglycerol in ray parenchyma cells [60, 61] and are utilized by plants as energy and carbon resources that fuel plant biomass production [58, 59, 62]. The present study revealed that increased amounts of lipid droplets in the transgenic *DX15::ScWS* lines were deposited in ray cells. Greater triacylglycerol content in the transgenic lines corresponded to greater expression levels of *DX15* but more studies would be necessary to confirm a direct link between *DX15* promoter activity and lipid content. Since our biochemical data indicate increases in triacylglycerol, it is reasonable to assume that the lipids droplets harbor triacylglycerol, alike storage lipids. In light of previous studies reporting increased wax ester concentrations in *ScWS*-expressing plants [45, 54–56], the present results were unexpected. The lack of primary alcohols, which are required—together with fatty acids—for wax ester production [52], may have prevented wax ester formation in wood. The metabolic shift toward increased triacylglycerol might have been due to the bifunctional activity of ScWS, which linked fatty acids in lieu of a primary fatty alcohol to a glycerol molecule, as has been shown for bacterial wax synthases [63–65]. In other studies, a significant enhancement of wax ester production was obtained after co-expression of *ScWS* with *FATTY ACID REDUCTASE* [24]. Therefore, it will be worthwhile to test whether poplar transformation with *FATTY ACID REDUCTASE* under the *DX15* promoter can induce the production of primary fatty alcohols in wood and lead to wax ester formation when co-expressed with *ScWS.*.

### Improved water repellency of poplar wood

A main hurdle toward technological wood usage is its dimensional instability due to swelling and shrinking [66]. Water uptake further renders wood susceptible to fungal-mediated degradation [67, 68]. Our study represents an important step toward wood improvement since we demonstrated that the *DX15::ScWS* lines had increased wood hydrophobicity, as indicated by decreased passive water uptake (− 25%) and decreased wettability by water droplets on the wood surface. Our study highlights that genetic engineering is a feasible technology for improving wood properties, potentially expanding the range of industrial applications where greater wood hydrophobicity is needed, for example, in fiber boards.

To date, wood has been chemically modified to increase its water repellency and durability. For example, Gordobil et al. [69] demonstrated that the esterification of lignin with long aliphatic carbon chains (12C) created a hydrophobic lignin derivative, which could be used as a protective agent for wood products. Furthermore, green technologies, often using vegetable oils, are underway to replace petrochemical products, e.g., in packaging materials with novel plant-based products [70]. For example, coating cellulose with tall oil (a side-product mainly obtained by processing pine wood) drastically decreased the water permeance (− 35%) and increased the elasticity of cellulose films [71]. Hydrophobization is used in the pulping process to increase paper quality, for the production of hemicellulose-based carrier materials for biomedical usage or as an antimicrobial treatment of wood products [72–74]. These examples highlight the wide spectrum of possible applications and imply benefits of biotechnological wood hydrophobization for industrial wood processing. Although our study revealed lipid droplet accumulation in wood, it remains unclear how increased water repellency was achieved. One possibility is that the lipid droplets present in rays of the intact xylem were distributed on the wood surface during wood processing. Other options could be the incorporation of lipids into cell walls, as observed, for example, in winter rye cell walls or cotton fibers [75, 76], or after the deregulation of wax biosynthesis by the overexpression of *AtMYB41* in *Arabidopsis thaliana* and *Nicotiana benthamiana* [77]. Further studies are necessary to clarify these possibilities.

The next step to achieve progress toward applications will require the growth of poplars under field conditions. These studies are difficult—if not impossible—to perform under the present restrictive legislation in Europe for genetically modified organisms [78]. Nevertheless, our study demonstrates the great potential of forest biotechnology for tree amendment. Our biotechnological approach offers potential economic benefits by reducing reliance on chemical treatments and contributes to sustainability efforts.

### Poplars transformed with DX15::ScWS have greater wood density

An unexpected result of our study was that the wood-specific promoter *DX15*, which naturally controls the expression of the *P. trichocarpa FLA-like AGP15,* caused increased production of fiber cell walls, thereby increasing wood density. Since we observed increased levels of *FLA-like AGP15* transcript abundances in the transgenic *DX15::ScWS* lines, a plausible explanation may be that transformation with the *DX15* sequence influenced cellulose deposition in fibers by recruiting *FLAs*. FLAs play a vital role in maintaining the structural integrity and functionality of the xylem by interacting with other cell wall components to reinforce cell walls and prevent collapse under tension or pressure [79]. In poplar, FLAs have been associated with cell wall deposition and tension wood formation for a long time [80–82], but the upregulation of *FLA*s by the overexpression of the *DX15* promoter in the absence of the structural gene itself is a novel result.

FLAs are members of a large gene family consisting of 35 genes in poplar [83, 84]. The nomenclature of these genes is inconsistent; the *P. trichocarpa* gene, whose promoter region was used here, was termed *FLA10* by [83] and *FLA-like AGP15* in PlantGenie (accessed 28th April 2024) and in JGI *P. trichocarpa* v3.1 but *FLA-like AGP12* in JGI *P. trichocarpa* v4.1. *PtFLA-like AGP15* has high homology to Arabidopsis *AtFLA11* and *AtFLA12* [83], which regulate secondary cell wall deposition [85–87]. *AtFLA11* and *AtFLA12* respond to mechanical stimuli, thereby influencing cell wall tensile strength and stiffness [85, 87]. To date, many transgenic studies succeeded in manipulation of wood traits in poplars [7]. Overexpression of specific transcription factors was an efficient strategy (e.g. *PeNAC122* [88], *PeCOB11* [89], *MYB46* in combination with GA20 oxidase [90]). Our study revealed that expression of the *DX15::ScWS* construct resulted in a fiber phenotype similar to that of *proFLA11::HIS-YFP-FLA11* overexpression in *Arabidopsis thaliana,* with substantially thicker cell walls than those of the WT [87]. In contrast to *Arabidopsis thaliana* [87], dwarfing of the poplar stem was not observed in our study. The mechanism by which FLAs affect cellulose production and cell wall deposition is still unclear [84]. In the promoter regions of *AtFLA11* and *AtFLA12,* transcription factor-binding sites for the regulation of secondary cell wall composition, such as MYB and DOF domains, are present [87, 91]. These transcription factors can act as negative regulators of other genes involved in xylem development [92]. DOF and MYB motifs were also abundant in the *DX15* promoter. Whether *DX15* also has intricate, cross-coordinating functions in poplar wood needs further analysis.

## Conclusion

The novelty and significance of this study lies in its pioneering exploration of the use of the *DX15* promoter in conjunction with the Jojoba wax ester synthase gene, *ScWS.* We found that a decrease in wood wettability was most likely achieved by increasing the production of lipid droplets within the wood structure. The improvement in wood hydrophobicity, a prerequisite for many industrial applications, potentially offers a sustainable and environmentally friendly alternative to the currently applied chemical treatments. The accumulation of neutral lipids in wood may offer additional opportunities for the green production of industrial commodities.

We also found increased wood density as the result of increased deposition of secondary cell wall layers in fiber cells. Wood density is an important technological property associated with increased mechanical strength and stiffness, rendering wood more resistant to bending, compression, and tension forces. Our results suggest that the promoter of *FLA-like AGP15* may constitute a regulatory circuit between FLAs and wood production. Further molecular studies of the promoter region are necessary to elucidate the function of *DX15* and illuminate how wood formation is intertwined with lipid metabolism. Overall, our study underscores the promising potential of the use of *DX15* promoter-driven transgenic lines as a novel strategy for obtaining wood with enhanced water resistance and durability in conjunction with increased wood density.

## Supplementary Information

Additional file 1.

## Data Availability

The datasets supporting the conclusions of this article are available in the repository Figshare under 10.6084/m9.figshare.28546739.
