# Supplementary material for: Ectopic expression of wax ester synthase under a wood-specific promoter enhances cell wall production and wood hydrophobicity
Source: Biotechnol Biofuels Bioprod. 2025 Jun 22;18:66. doi: 10.1186/s13068-025-02667-w (PMC12182671; doi:10.1186/s13068-025-02667-w)
Supplement: Supplementary file 1 — Additional file 1. [file 13068_2025_2667_MOESM1_ESM.pdf]

## Supporting Materials

# Ectopic expression of wax ester synthase under a wood-specific promoter enhances cell wall production and wood hydrophobicity

Ashkan Amirkhosravi, Gerrit-Jan Strijkstra, Alisa Keyl, Linus Heydenreich, Cornelia Herrfurth, Ivo Feussner, Andrea Polle

**Supplemental Figure S1**. Alignment of the sequences of the *DX15* region of *FLA-like AGP15* from *P. nigra* × *maximowiczii* (gray underlay, published by Ko et al. 2012) and from Potri.009G012200 of *P. trichocarpa.*

**Supplemental Figure S2.** Motif analysis of the *DX15* promoter sequence via the PlantCARE database.

**Supplemental Figure S3.** PCR screening of *DX15::ScWS*-transformed plants (a) and the expression vector construct used to transform Agrobacterium (b).

**Supplemental Figure S4.** Scheme of the stem sampling procedure.

**Supplemental Figure S5.** Stomatal morphology, stomatal length, and stomatal density of the wild-type and *DX15::ScWS* poplar lines.

**Supplemental Figure S6.** Typical wood cross sections of wild-type (WT), *DX15::ScWS1*, and *DX15::ScWS2* poplar lines and their ray areas and fractions of cell walls.

**Supplemental Figure S7**. Composition and quantities of cuticular waxes on the bark of the wild-type (WT, a), *DX15*::*Sc*WS1 (b) and *DX15*::*Sc*WS2 (c) *P*. x *canescens* lines.

**Supplemental Table S1.** List of the primers and Potri numbers for the genes used for cloning and for expression analyses via qRT–PCR.

**Supplemental Table S2.** Comparative analysis and prediction of *cis*-acting elements within the *DX15* region across multiple species.

**Supplemental Table S3.** Growth, gas exchange and wood properties of *P. canescens* WT and transgenic *DX15::ScWS* lines (*DX15::ScWS1* and *DX15::ScWS2*).

**(a)**

```
Ko DX15          TTCCCCCTTTTGGTTCAATGCCTTTTATTCTTCCAAAATTATTTCA-TATTTTGTATCCGGAGGA-CATA     68
P. trichocarpa   TTCCCCCTTTTGGTTCAATGCCTTTTATTCTTCCAAATTAATTTCAATATTTTGTATCCGGAGGAACATA     70

Ko DX15          TTTGTTTCAAAAGGTGTCAGAAAATCAAAGCCCATTGAAAATATATAAACATATATAGATATAAAAACTC    138
P. trichocarpa   TTTGTTTCAAAAGGTGTCAGAAAATCAAAGCCCATTGAAAATATATAAACATATATAGATATAAAAACTC    140

Ko DX15          AAGGGTTCATTCCAAAATATAAGAACAAACTGATTGAATTAATTTGTTATTTTAAGAACACTGTCTATAT    208
P. trichocarpa   AAGGATTCATTCCAAAATATAAGAACAAACTGATTGAATTAATTTGTTATTTTAAGAACACTGTCTATAT    210

Ko DX15          GTTTATATAGTGGGAGGTAGTGTTTTTTAAATCATATACTAACTTATTATAAAAATAAATCATAAAAAAG    278
P. trichocarpa   GTTTATATAGTGGTAGGTAGTGTTTTTAAAATCATATACTAACTTATTATAAAAATAAATCATAAAAAAG    280

Ko DX15          GAACCTCAAGCATCCCCTGGTAAGCTCGTATGTAGGAATACTCGGAGATCAAATGTCCGAATGTCAAATG    348
P. trichocarpa   GAACCTCAAGCATCCCCTGGTAAGCTCGTATGTAGGAATACTCGGAGGTCAAATGTCCGAATGTCAAATG    350

Ko DX15          TTAAGGCAAGTGAAATATCCCTGACTTTTTAGCAAGCAAATTGTTGAGTAGCTAAAATGAATTATTTTAA    418
P. trichocarpa   TTAAGGCAAGTGAAATATCCCTGACTTTTTAGCAAGCAAATTGTTGAGTAGCTAAAATGAATTATTTTAA    420

Ko DX15          TATTTTTAAATCATTTTAATATATTAATATTAAAAAAAATTAAATATTTTTTTTAATACATTTTCAATAA    488
P. trichocarpa   TATTTTCAAATCATTTTAATATATTAATATTAAAAAAAATTAAATATTTTTTTTAATACATTTTCAATAA    490

Ko DX15          CAAACACTTTAAAATATAATCTTTGTCACACTCTTAAACAGTAACAGCAGAAAGCATATGTGAGTGATAT    558
P. trichocarpa   CAAACACTTTAAAATATAATCTTTGTCACACTCTTAAACAGTAACAGCAGAAAGCATATGTGAGTGATAT    560

Ko DX15          AGCTATAGTTGCTGTTTGACACGGACAATCTCCATCTAAATTCATGAATAATAAAGTTTTGCCTACACAC    628
P. trichocarpa   AGCTATAGTTGCTGTTTGACACGGACAATCTCCATCTAAATTCATGAATAATAAAGTTTTGCCTACACAC    630

Ko DX15          CCACTTGAAATCTCCTCCTAGTTTTCCTGATTTGCCATGCTAACTACAAGAACAAGATGCTAGCTAGTAT    698
P. trichocarpa   CCACTTGAAATCTCCTCCTAGTTTTCCTGATTTGCCATGCTAACTACAAGAACAAGATGCTAGCTAGTAT    700

Ko DX15          CTTGTTCTGTCTCTCGCTCTCTCTCTATCTCTCCAGTTGATAGTTGATAGTTGATAGTTGATAGCTGATA    768
P. trichocarpa   CTTGTTCTGTCTCTCGCTCTCTCTCTACCTCTCCAGTTGATAGTTGATAGTTGATAG-------CTGATA    763

Ko DX15          CCCTCCCACCTTTCCCAGAAAGATGATTGAGGAACTAGTCACTGTGTTCGTGTAACTAATACTGTTCATG    838
P. trichocarpa   CCCTCCCACCTTTCCCAGAAAGATGATTGAGGAACTAGTCACTGTGTTCGTGTAACTAATACTGTTCATG    833

Ko DX15          GCACCTAACTTGATCCTCTCTTCACCAGACCACTATAAAAACCCTATCTGTCCTCCTCATAATCATATCA    908
P. trichocarpa   GCACCTAACTTGATCCTATCTTCACCAGACCACTATAAAAACCCTATCTGTCCTCCTCATAATCATATCA    903

Ko DX15          CTACACCCAACACTTCTGCAAGCACAACTCCATTCAAGAACATCAAGAGTATAGGCCGCCGCTGCAACAA    978
P. trichocarpa   CTACACCCAACACTTCTGCAAGCACAACTCCATTCAAGAACATCAAGAGTATAGGCCGCCGCTGCAACAA    973

Ko DX15          AACAGCACTCCTAGCTACTTCAAGATGAGGCCACAATCTTTCATCTT                          1025
P. trichocarpa   AACAGCACTCCTAGCTACTTCAAGATGAGGCCACAATCTTTCATCTT                          1020
```

**(b)**

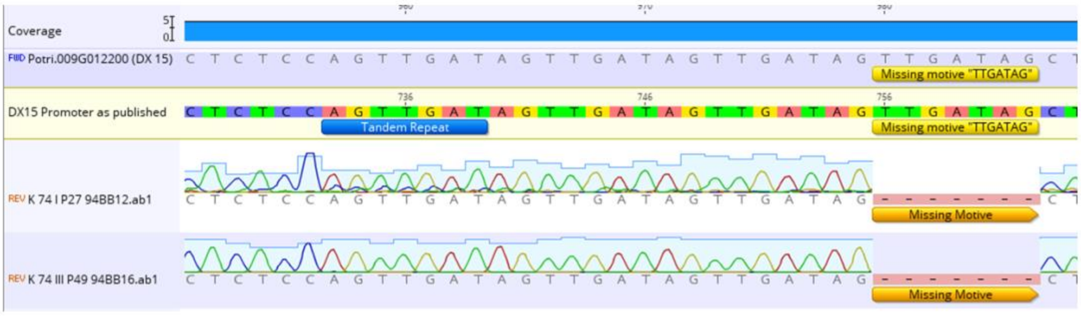


**Supplemental Figure S1**. Alignment of the sequences of the *DX15* region of *FLA-like AGP15* from *P. nigra* x *maximowiczii* (gray underlay, published by Ko et al. 2012) and from Potri.009G012200 of *P. trichocarpa* (a) and a close-up of DX15 for two of three individuals of *P. trichocarpa* (var. Nisqualy) in the region of the repeated motif 3'-TTGATAG-5' (b). The *DX15* promoter as shown in (a) from *P. trichocarpa* was cloned in this study. The sequences show 98.9% similarity with that published by Ko et al. (2012). At position 733, the motif TTGATAG is repeated four-times in *P. nigra* x *maximowiczii* but only three-times in *P. trichocarpa*.

```
>PlantCARE_11068
+ TCCCCCTTTT GGTTCAATGC CTTTTATTCT TCCAAATTAA TTTCAATATT TTGTATCCGG AGGAACATAT
- AGGGGGAAAA CCAAGTTACG GAAAATAAGA AGGTTTAATT AAAGTTATAA AACATAGGCC TCCTTGTATA

+ TTGTTTCAAA AGGTGTCAGA AAATCAAAGC CCATTGAAAA TATATAAACA TATATAGATA TAAAAACTCA
- AACAAAGTTT TCCACAGTCT TTTAGTTTCG GGTAACTTTT ATATATTTGT ATATATCTAT ATTTTTGAGT

+ AGGATTCATT CCAAAATATA AGAACAAACT GATTGAATTA ATTTGTTATT TTAAGAACAC TGTCTATATG
- TCCTAAGTAA GGTTTTATAT TCTTGTTTGA CTAACTTAAT TAAACAATAA AATTCTTGTG ACAGATATAC

+ TTTATATAGT GGTAGGTAGT GTTTTTAAAA TCATATACTA ACTTATTATA AAAATAAATC ATAAAAAAGG
- AAATATATCA CCATCCATCA CAAAAATTTT AGTATATGAT TGAATAATAT TTTTATTTAG TATTTTTTCC

+ AACCTCAAGC ATCCCCTGGT AAGCTCGTAT GTAGGAATAC TCGGAGGTCA AATGTCCGAA TGTCAAATGT
- TTGGAGTTCG TAGGGGACCA TTCGAGCATA CATCCTTATG AGCCTCCAGT TTACAGGCTT ACAGTTTACA

+ TAAGGCAAGT GAAATATCCC TGACTTTTTA GCAAGCAAAT TGTTGAGTAG CTAAAATGAA TTATTTTAAT
- ATTCCGTTCA CTTTATAGGG ACTGAAAAAT CGTTCGTTTA ACAACTCATC GATTTTACTT AATAAAATTA

+ ATTTTCAAAT CATTTTAATA TATTAATATT AAAAAAAATT AAATATTTTT TTTAATACAT TTTCAATAAC
- TAAAAGTTTA GTAAAATTAT ATAATTATAA TTTTTTTTAA TTTATAAAAA AAATTATGTA AAAGTTATTG

+ AAACACTTTA AAATATAATC TTTGTCACAC TCTTAAACAG TAACAGCAGA AAGCATATGT GAGTGATATA
- TTTGTGAAAT TTTATATTAG AAACAGTGTG AGAATTTGTC ATTGTCGTCT TTCGTATACA CTCACTATAT

+ GCTATAGTTG CTGTTTGACA CGGACAATCT CCATCTAAAT TCATGAATAA TAAAGTTTTG CCTACACACC
- CGATATCAAC GACAAACTGT GCCTGTTAGA GGTAGATTTA AGTACTTATT ATTTCAAAAC GGATGTGTGG

+ CACTTGAAAT CTCCTCCTAG TTTTCCTGAT TTGCCATGCT AACTACAAGA ACAAGATGCT AGCTAGTATC
- GTGAACTTTA GAGGAGGATC AAAAGGACTA AACGGTACGA TTGATGTTCT TGTTCTACGA TCGATCATAG

+ TTGTTCTGTC TCTCGCTCTC TCTCTACCTC TCCAGTTGAT AGTTGATAGT TGATAGCTGA TACCCTCCCA
- AACAAGACAG AGAGCGAGAG AGAGATGGAG AGGTCAACTA TCAACTATCA ACTATCGACT ATGGGAGGGT

+ CCTTTCCCAG AAAGATGATT GAGGAACTAG TCACTGTGTT CGTGTAACTA ATACTGTTCA TGGCACCTAA
- GGAAAGGGTC TTTCTACTAA CTCCTTGATC AGTGACACAA GCACATTGAT TATGACAAGT ACCGTGGATT

+ CTTGATCCTA TCTTCACCAG ACCACTATAA AAACCCTATC TGTCCTCCTC ATAATCATAT CACTACACCC
- GAACTAGGAT AGAAGTGGTC TGGTGATATT TTTGGGATAG ACAGGAGGAG TATTAGTATA GTGATGTGGG

+ AACACTTCTG CAAGCACAAC TCCATTCAAG AACATCAAGA GTATAGGCCG CCGCTGCAAC AAAACAGCAC
- TTGTGAAGAC GTTCGTGTTG AGGTAAGTTC TTGTAGTTCT CATATCCGGC GGCGACGTTG TTTTGTCGTG

+ TCCTAGCTAC TTCAAGATGA GGCCACAATC TTTCATCT
- AGGATCGATG AAGTTCTACT CCGGTGTTAG AAAGTAGA
```

**Supplemental Figure S2**. Motif analysis in the *DX15* promoter sequence using the PlantCARE database. The *DX15* promoter sequences obtained by Sanger sequencing were uploaded to PlantCARE (https://bioinformatics.psb.ugent.be/webtools/plantcare/html/). PlantCARE uses known consensus sequences and motifs to predict the presence of cis-acting elements within the input promoter sequences. Here, 17 different sites were predicted. Blue colors: Blue box, Yellow color: ACE, Maron color:AT~TATA-box, Red color: Box4, Light green: CAAT-box, Crayola blue: ERE, Light pink: GATA-motif, Pink: MBS, Cyan: MYC, Deep sky blue: MYB, Iceberg: P-box, Pink Rose: STRE, Dark yellow: TATA-box, Orange: unnamed_4, Magenta: W box, Light yellow: WR3. light Green: DOF motifs. The positions and species comparisons are shown in Supplement Table S2.

**(a)**

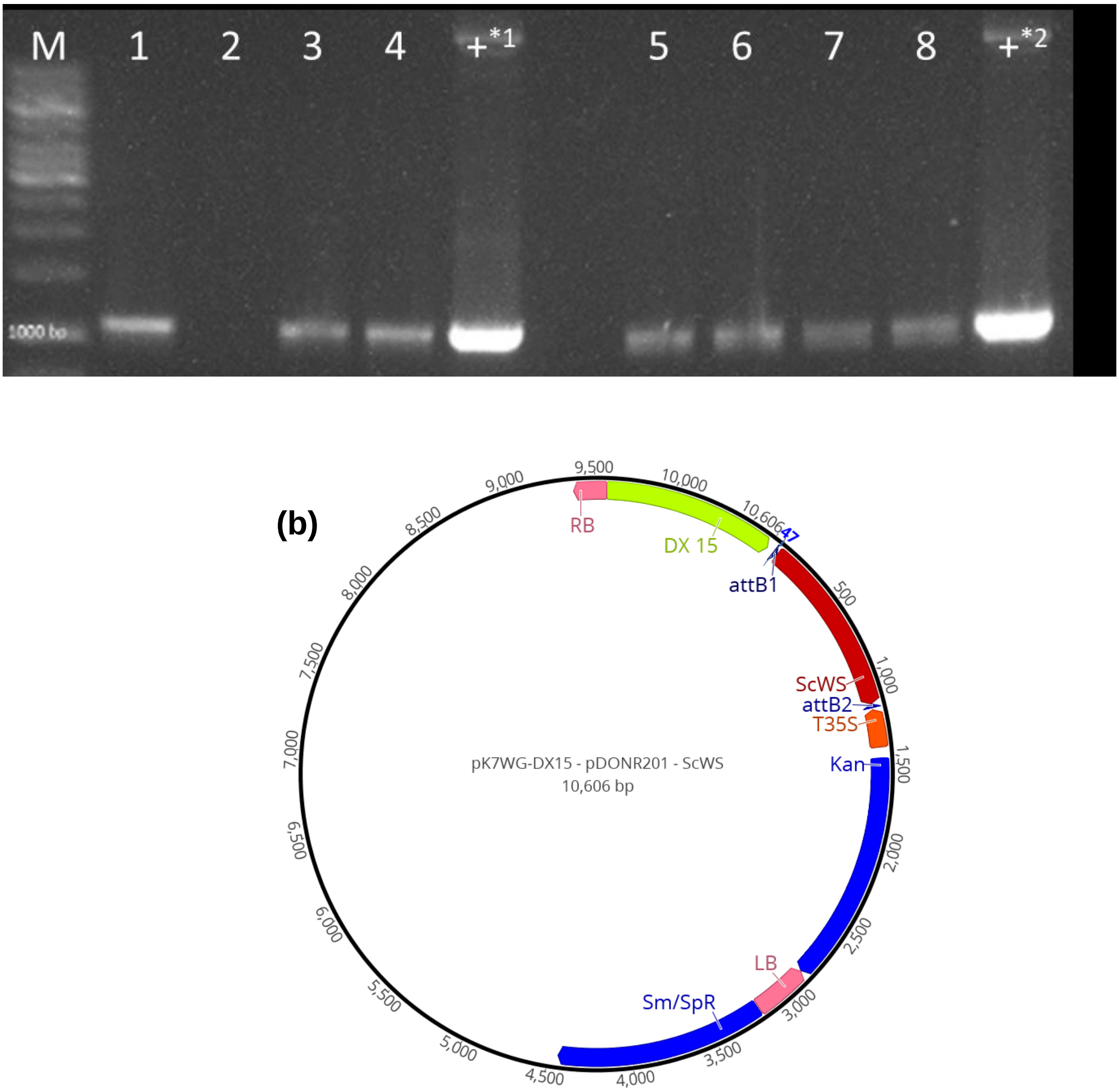


**Supplemental Figure S3.** PCR screening of *DX15::ScWS* transformed *P.* x *canescens* (a) and expression vector construct used to transform Agrobacterium (b). The PCR screening involved detection of *Sc*WS (lanes 1-4) using ScWS specific primers and the *DX15* promoter (lanes 5-8) utilizing *DX15* promoter primer pairs. In the gel image, M represents the marker lane. Lane 1 shows *DX15*::*Sc*WS1, Lane 2 indicates a negative result, leading to the exclusion of the corresponding line from further experiments. Lane 3 represents *DX15*::*Sc*WS2, and Lane 4 shows *DX15*::*Sc*WS3. Lane 5 shows *DX15*::*Sc*WS1, Lane 6 indicates an expelled line, Lane 7 represents *DX15*::*Sc*WS2, and Lane 8 shows *DX15*::*Sc*WS3. Additionally, +*1 denotes the positive control for *Sc*WS, and +*2 represents the positive control for *DX15*.

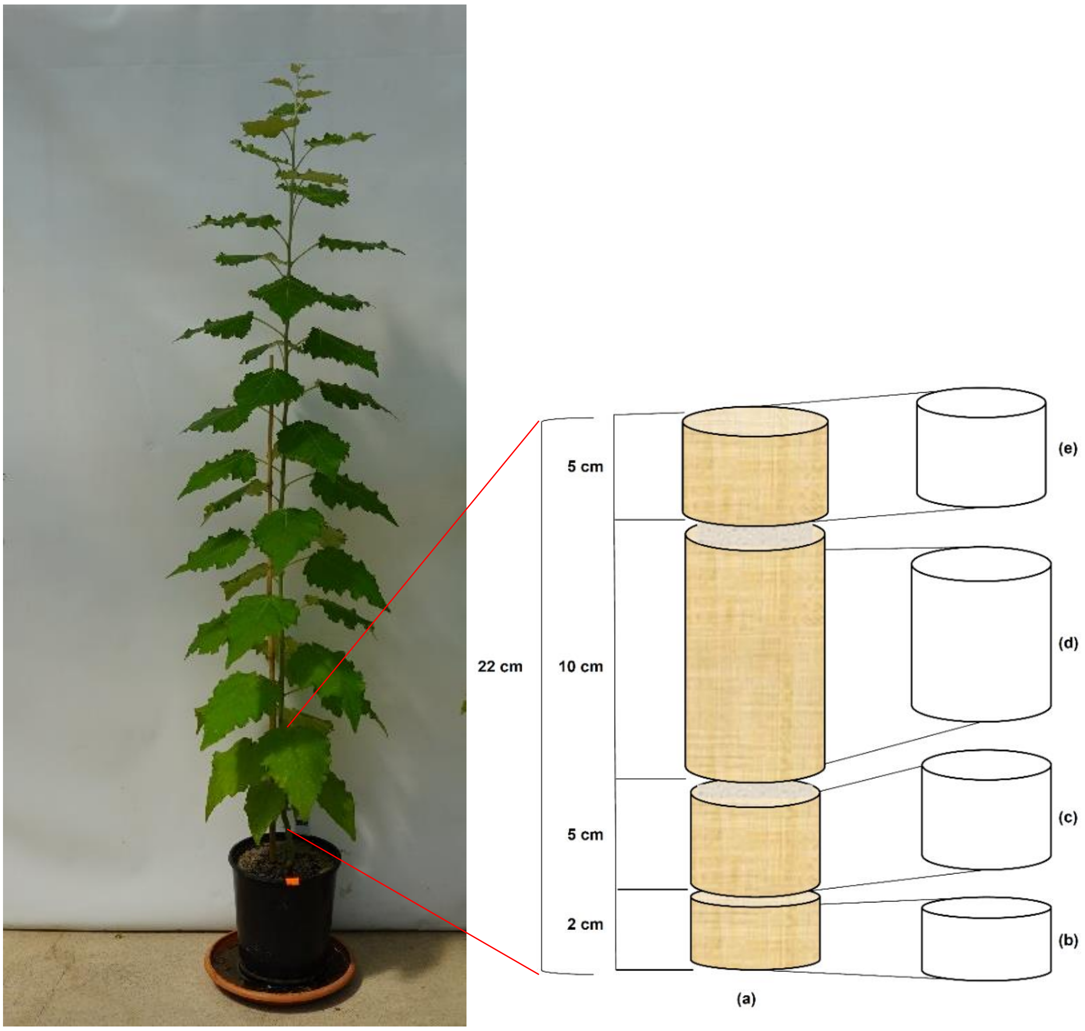


**Supplemental Figure S4.** Scheme for the stem sampling procedure. A stem segment of 22 cm from the bottom of 100-day-old poplars (a) was cut into different parts used for distinct analyses: The first 2 cm (b) at the stem bottom, where the diameter of the plants had been measured, was used for wood anatomical analysis and fixed in FAE solution. Section (c) was divided into bark and wood. The bark was used for surface wax ester analysis and the wood shock-frozen in liquid nitrogen and stored -80°C. Part (d) was 10 cm long, debarked und used for wood properties (wood density, wood water uptake, wood hydrophobicity). Stem section (e) was debarked. The fresh wood surface was immediately scraped off; the slurry (=developing xylem) was shock-frozen in liquid nitrogen and stored at -80°C for molecular analyses.

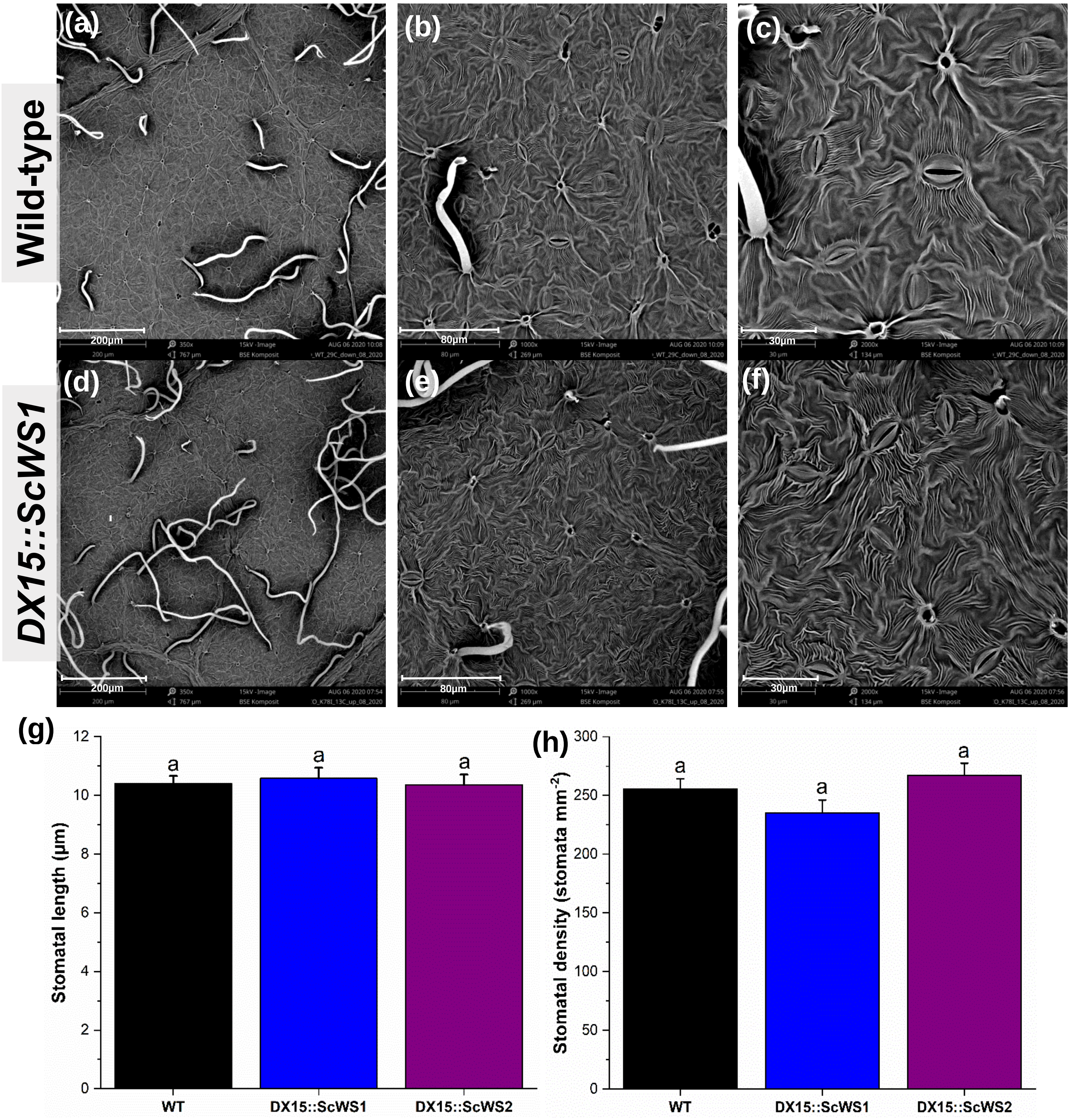


**Supplemental Figure S5.** Stomatal morphology of the wild-type (WT a,b,c) and line *DX15::Sc*WS1 (c,d,e), stomatal lengths (g) and stomatal densities (h). Panels a to e depict examples for the lower leaf surface at 350x magnification (a,d), 1000x magnification (b,e), and 2000x magnification (c,f) viewed under a scanning electron microscope as described by Amirkhosravi et al. (2024). Stomatal lengths and densities were measured on 4 to 5 individual samples per line. Data show means (± SE). No significant differences were observed among means at $p \leq 0.05$ (ANOVA).

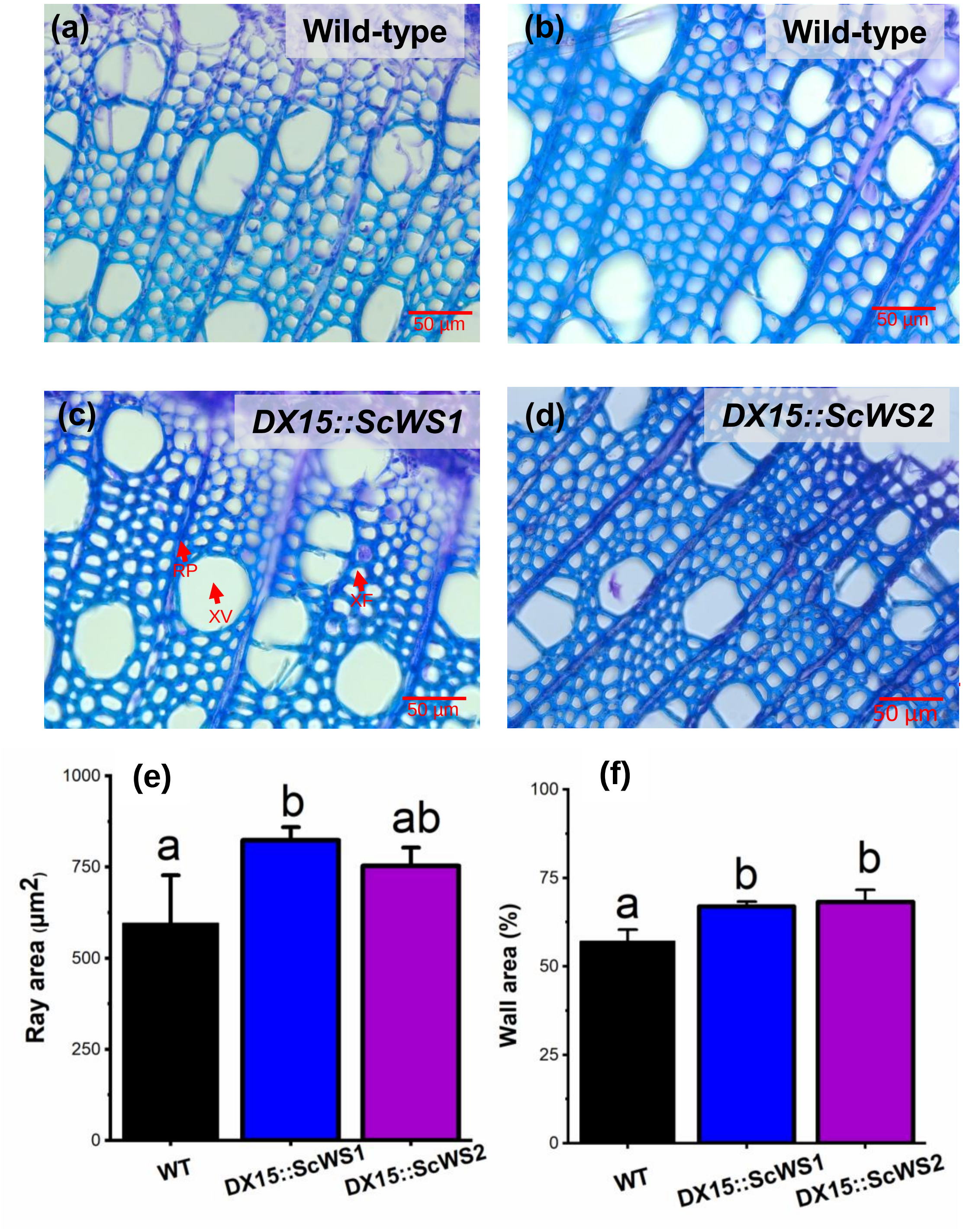


**Supplemental Figure S6.** Typical wood cross sections of wild-type (WT a,b), *DX15::ScWS1* (c), and *DX15::ScWS2* (d) poplar lines and their ray area (e) and fraction of cell walls (f). The cross sections were stained with toluidine blue. RP: Ray Parenchyma, XV: Xylem vessel, XF: Xylem fiber. Data in (e) and (f) show means (n = 6 of individual plants per line, ± SE). Different letters indicate significant differences among the lines at p ≤ 0.05 (ANOVA, post-hoc Tukey test)

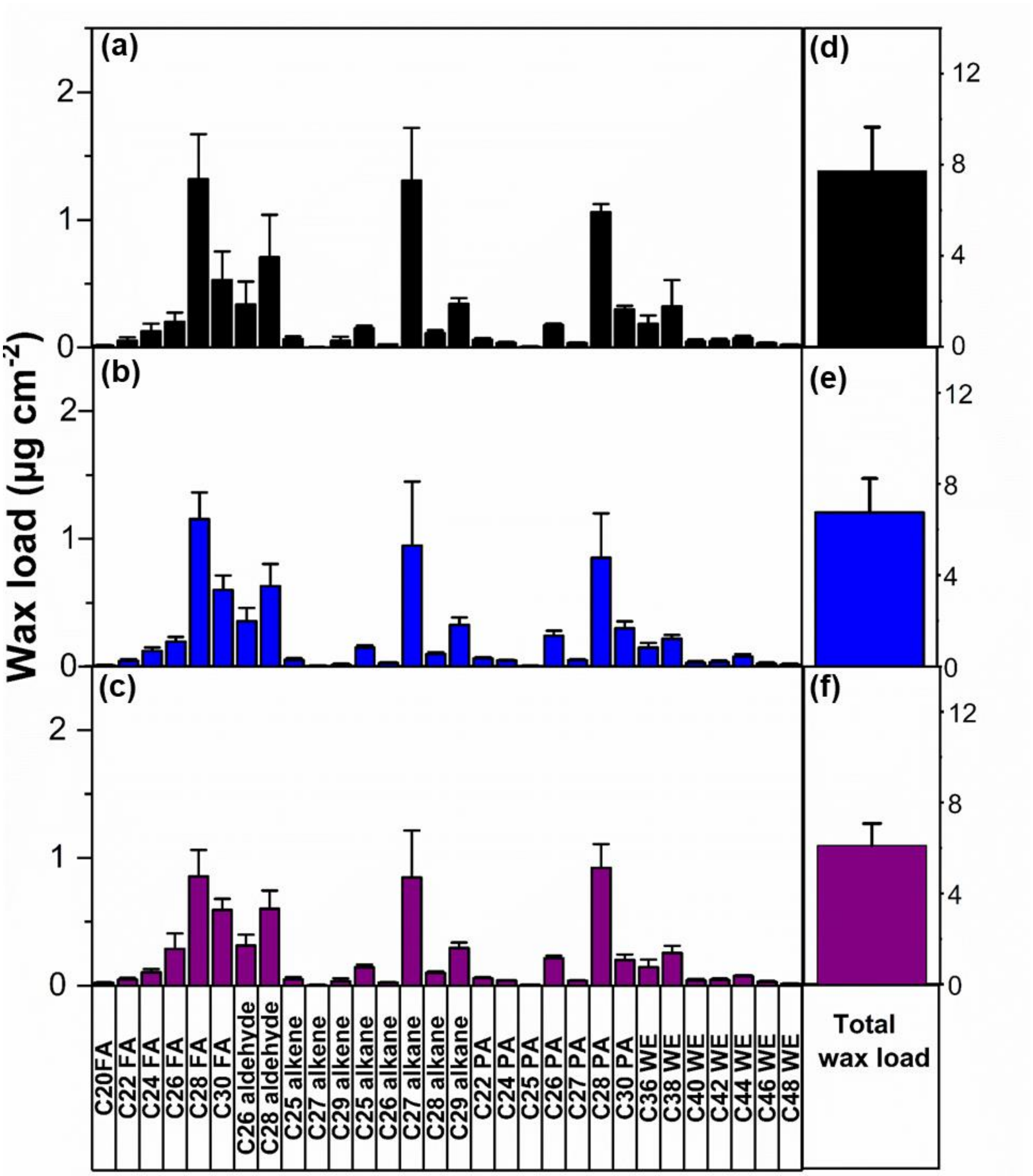


**Supplemental Figure S7.** Composition and quantities of cuticular waxes on the bark of wild-type (WT, a), *DX15*::*Sc*WS1 (b) and *DX15*::*Sc*WS2 (c) *P*. x *canescens* lines. $C_n$ indicates the number of aliphatic C atoms in the following components: fatty acids (FA), aldehydes, alkanes, alkenes, primary alcohols (PA), and wax esters (WE). Panels (d), (e), and (f) represent the total wax load, calculated as the sum of all wax substance classes. The data showed high variation per substance class and were therefore log-transformed to achieve normal distribution for ANOVA. Significant differences at $p < 0.05$ were not detected ($p = 0.205$). Bars represent means (n = 5 or 7 individual plants per line, ± SE).

**Supplemental Table S1.** List of the primers and Potri numbers for genes used for cloning and for expression analyses by qRT PCR.

| Gene name | Potri Nr. | Sequence |
|---|---|---|
| ***Sc*WS** | Cloning | FOR: attL1- GGGGACAAGTTTGTACAAAAAAGCAGGCTTAGTCGACATGGAGGTGGAG |
| | | REV: attL2- GGGGACCACTTTGTACAAGAAAGCTGGGTTCTCACCACCCCAACAAACC |
| ***p*DONR201** | Cloning | FOR: TCGCGTTAACGCTAGCATGGATCTC |
| | | REV GTAACATCAGAGATTTTGAGACAC |
| ***Sc*WS** | qPCR | FOR: ATG GTG GTG AAG AAG GCG G |
| | | REV: TCC AGT CAC CAT CAC GAA CC |
| **pK7WG** | Cloning | FOR:GCGGGAAACGACAATCTG |
| | | REV: TTGCGGACTCTAGCATGG |
| **DX15 HindIII** | Cloning | FOR. TTTTTAAGCTTTTCCCCCTTTTGGTTCAATGCCTTTTATTCTTCC |
| | | |
| **DX15 Sac I** | Cloning | |
| | | REV: TTTTTGAGCTCAAGATGAAAGATTGTGGCCTCATCTTGAAGTAGC |
| **DX5 HindIII** | Cloning | FOR: TTTTTAAGCTTGGGGCAGATGATACCTTGATACTTGGACTAGG |
| | | |
| **DX5 Sac I** | Cloning | |
| | | REV: TTTTTGAGCTCCTTTGGTTTCCTCTTATGCCCATATATTGCACACC |
| **DX15** | Cloning | FOR. 5'-TTC CCC CTT TTG GTT CAA TG-3' |
| | | REV: 5'-GAG GCC ACA ATC TTT CAT CTT-3' |
| **DX5** | Cloning | FOR: 5'-GGG GCA GAT GAT ACC TTG ATA CTT-3' |
| | | REV. 5'-TGG GCA TAA GAG GAA ACC AAA G-3' |
| **FLA-like AGP15** | qPCR Potri.009G012200 | FOR. CTCTAAGCGATGAAGACAAGAC |
| | | REV: TCCTTACAGGGTTACTCACAG |
| ***Ptr*PPR_2** | Potri.012G141400 | FOR: ATCGTTCCAAGTCAAGTATGTG |
| | | REV: TCAAGGGAGCAACTTTACAG |
| ***Ptr*Rpp14** | Potri.015G001600 | FOR: GCAATGTGAGGAGTTTAGGG |
| | | REV: TATTAAATGTCTGTGCTGTAGTGTG |

**Supplemental Table S2.** Comparative analysis of predicted of cis-acting elements within the DX15 region across multiple species. The analyses were conducted with PLANTCARE. Mapping of motif names to the colored sequences represented in Supplement Figure S2. Each color code corresponds to a specific cis-acting element.

| Site name | Organism | Position | Strand | Matrix Score | Sequence | Function |
|---|---|---|---|---|---|---|
| **Blue** | *Populus trichocarpa* | 63 | - | 4 | motif_sequence | Short_function |
| *ACE* | *Petroselinum crispum* | 248 | + | 9 | CTAACGTATT | cis-acting element involved in light responsiveness |
| ***AT~TATA-box*** | *Arabidopsis thaliana* | 211 | - | 8 | TATATAAA | ND |
| | *Arabidopsis thaliana* | 111 | + | 6 | TATATA | ND |
| | *Arabidopsis thaliana* | 121 | + | 6 | TATATA | ND |
| | *Arabidopsis thaliana* | 213 | + | 6 | TATATA | ND |
| ***Box 4*** | *Petroselinum crispum* | 36 | + | 6 | ATTAAT | part of a conserved DNA module involved in light responsiveness |
| | *Petroselinum crispum* | 442 | + | 6 | ATTAAT | part of a conserved DNA module involved in light responsiveness |
| | *Petroselinum crispum* | 177 | + | 6 | ATTAAT | part of a conserved DNA module involved in light responsiveness |
| ***CAAT-box*** | *Nicotiana glutinosa* | 15 | + | 4 | CAAT | common cis-acting element in promoter and enhancer regions |
| | *Pisum sativum* | 181 | - | 5 | CAAAT | common cis-acting element in promoter and enhancer regions |
| ***ERE*** | *Nicotiana glutinous* | 497 | - | 8 | ATTTTAAA | plays a role in the defense response against bacterial pathogens |
| ***GATA-motif*** | *Arabidopsis thaliana* | 846 | - | 7 | GATAGGA | part of a light responsive element |
| | *Pisum sativum* | 874 | - | 7 | GATAGGG | part of a light responsive element |
| ***MBS*** | *Arabidopsis thaliana* | 733 | - | 6 | CAACTG | binding site involved in drought inducibility |
| ***MYC*** | *Arabidopsis thaliana* | 329 | - | 6 | CATTTG | regulating leaf senescence, root elongation, seed production |
| | *Arabidopsis thaliana* | 344 | - | 6 | CATTTG | response to drought and ABA signals |
| ***MYB*** | *Arabidopsis thaliana* | 733 | - | 6 | CAACTG | response to drought and ABA signals |
| ***P-box*** | *Oryza sativa* | 5 | + | 7 | CCTTTTG | gibberellin-responsive element |
| ***STRE*** | *Arabidopsis thaliana* | 3 | - | 5 | AGGGG | ND |
| ***TATA-box*** | *Brassica napus* | 110 | + | 6 | ATATAT | core promoter element around -30 of transcription start |
| | *Brassica oleracea* | 128 | + | 6 | ATATAA | core promoter element around -30 of transcription start |
| | *Arabidopsis thaliana* | 113 | + | 4 | TATA | core promoter element around -30 of transcription start |
| | *Helianthus annuus* | 211 | + | 6 | TATAAA | core promoter element around -30 of transcription start |
| | *Oryza sativa* | 49 | - | 7 | TACAAAA | core promoter element around -30 of transcription start |
| | *Arabidopsis thaliana* | 846 | + | 9 | CCTATAAAA | core promoter element around -30 of transcription start |
| ***Unnamed__4*** | *Petroselinum Hortense* | 765 | + | 4 | CTCC | ND |
| ***W box*** | *Arabidopsis thaliana* | 326 | - | 6 | TTGACC | ND |
| ***WRE3*** | *Pisum sativum* | 763 | + | 6 | CCACCT | ND |
| ***DOF Motif*** | *Arabidopsis thaliana* | 77 | - | 5 | AAAAG | contribute to guard cell-specificity of AtMYB60 promoter |

ND = no detected function

**Supplemental Table S3**. Growth, gas exchange and wood properties of *P.* x *canescens* wild-type (WT) and the transgenic lines (DX15:*Sc*Ws1, DX15::*Sc*WS2). Plants were grown for 90 days in a growth chamber (experiment 2: n = 5 to 6, ± SE). Different letters indicate significant differences at $p < 0.05$ (ANOVA, post-hoc Tukey Test)

| Parameter | WT | SE | | DX15::*Sc*WS1 | SE | | DX15::*Sc*WS2 | SE | |
|---|---|---|---|---|---|---|---|---|---|
| Height (cm) | 83.1 | 4.4 | a | 98.2 | 4.3 | b | 91.4 | 3.9 | ab |
| Diameter (mm) | 7.4 | 0.3 | a | 7.9 | 0.4 | a | 7.7 | 0.3 | a |
| Plant dry biomass (g) | 29.0 | 3.3 | a | 30.3 | 3.1 | a | 27.7 | 1.9 | a |
| Whole plant leaf area ($cm^2$) | 2622 | 202 | a | 3041 | 390 | a | 3010 | 225 | a |
| Number of leaves | 38 | 2 | a | 48 | 3 | b | 47 | 3 | b |
| Photosynthesis (µmol $m^{-2}$ $s^{-1}$) | 5.78 | 0.14 | b | 5.21 | 0.25 | ab | 5.01 | 0.23 | a |
| Stomatal conductance (mmol $m^{-2}$ $s^{-1}$) | 1.08 | 0.07 | a | 1.04 | 0.03 | a | 0.99 | 0.03 | a |
| Wood water uptake (g $g^{-1}$ $day^{-1}$) | 2.72 | 0.17 | a | 1.37 | 0.02 | b | 1.38 | 0.03 | b |
| Wood density (g $cm^{-3}$) | 0.23 | 0.03 | a | 0.42 | 0.07 | b | 0.30 | 0.05 | ab |
